# Efficacy of Different Types of Physical Activity Interventions on Exercise Capacity in Patients with Chronic Obstructive Pulmonary Disease (COPD): A Network Meta-Analysis

**DOI:** 10.3390/ijerph192114539

**Published:** 2022-11-05

**Authors:** Susana Priego-Jiménez, Ana Torres-Costoso, María José Guzmán-Pavón, Patricia Lorenzo-García, María Isabel Lucerón-Lucas-Torres, Celia Álvarez-Bueno

**Affiliations:** 1 Hospital Virgen de la Luz, 16002 Cuenca, Spain; 2 Health and Social Research Center, Universidad de Castilla La Mancha, 16071 Cuenca, Spain; 3 Faculty of Physiotherapy and Nursing, University of Castilla La Mancha, 45071 Toledo, Spain; 4 Facultad de Enfermería de Cuenca, Edificio Melchor Cano, Universidad de Castilla-La Mancha, 16071 Cuenca, Spain; 5 Universidad Politécnica y Artística del Paraguay, Asunción 2024, Paraguay

**Keywords:** COPD, pulmonary rehabilitation, systematic review, meta-analysis, exercise capacity, active mind-body movements therapies, physical activity, 6MWT

## Abstract

Aim: A network meta-analysis (NMA) was performed to determine the effects on the exercise capacity, measured by the 6 MWT, of patients with COPD of (i) different physical activity interventions and (ii) supervised or unsupervised programs. Methods: A literature search was carried out from inception to April 2022. Randomized controlled trials of the effectiveness of physical activity on exercise capacity in patients with COPD were included. The risk of bias was assessed using the Cochrane Risk of Bias (RoB 2.0) tool, and the Grading of Recommendations, Assessment, Development, and Evaluation tool (GRADE) was used to assess the quality of the evidence. A pairwise meta-analysis for direct and indirect effects was carried out. Results: A total of 41 studies were included in this NMA. The highest effects were for urban training pulmonary rehabilitation (PR) programs (ES, 1.50; 95% CI: 0.46 and 2.55) versus the control group. For supervised and unsupervised PR and home-based PR programs, the highest effects were found for supervised PR (ES, 0.85; 95% CI: 0.46 to 1.23) versus the control group. Conclusions: PR implemented with urban circuit training should be considered the most effective strategy to improve exercise capacity in patients with COPD. Supervision of the programs improves exercise capacity.

## 1. Introduction

Chronic obstructive pulmonary disease (COPD) is currently defined as a respiratory disease characterized by persistent symptoms and chronic airflow limitation, whose main cause is tobacco. Airflow limitation usually manifests as dyspnea and is usually progressive. COPD often occurs with other respiratory symptoms, such as chronic cough accompanied or not by expectoration and is characterized by the presence of exacerbations and the frequent presence of comorbidities that can contribute to the severity in some patients [1]. In recent years, it has become one of the leading causes of morbidity and mortality worldwide [2,3], with a high cost to health care systems, due not only to its requirement for lifelong pharmacological treatments but also to the high number of exacerbations and hospitalizations per year.

Symptoms that characterize COPD include decreased exercise capacity and dyspnoea [4] as well as a lower level of physical activity compared to healthy controls [5]. Studies also show impaired peripheral muscle strength and/or resistance with a consequent negative effect on the exercise capacity of patients with COPD and a decreased quality of life. Skeletal muscle weakness has been shown to be an important predictor of exercise limitation in COPD [6]. In addition, loss of skeletal muscle mass has been described as a predictor of worse prognosis or mortality, independent of the degree of airway obstruction [7,8]. Finally, exercise intolerance is a major problem of COPD patients and is closely related to disability, being a stronger predictor of poor quality of life and survival than spirometry or oxygenation [9,10,11]. Exercise tolerance, measured as exercise capacity, has been described as one of the main factors in understanding the impact of COPD and the development of disease management methodologies for COPD patients [12]; decreased exercise tolerance is one of the key features related to a poor prognosis in patients with COPD.

In recent years, the evaluation of the exercise capacity of patients with COPD in clinical and research settings, including simple gait, has increased. The most widely used test, the 6 min walk test (6 MWT), measures the maximum distance covered at the highest possible intensity in 6 min [13,14,15] and is a better reflection of the patient’s functional capacity for daily physical activities than conventional pulmonary function tests [16,17,18]. The 6 MWT has been shown to have good reliability and validity for measuring functional capacity [13,19] and has been shown to be related to maximal exercise test parameters.

To improve functional capacity and dyspnoea, physical training is considered an essential component of pulmonary rehabilitation (PR) in patients with COPD [13,20]. However, there is still no consensus on the best training strategy or on the possible mechanisms [21] of improvement. Several types of exercise, such as resistance, muscular power combined with resistance, PR, yoga, or tai chi, have been described as effective in improving exercise capacity in patients with COPD, but their relative effectiveness has not been explored. Additionally, the supervision of exercise programs could affect the effectiveness of these interventions. Finally, the specific characteristics of physical activity programs (i.e., intensity, frequency, and dose) and the difficulty of performing exercise programs should be considered when analyzing the effect of exercise among patients with COPD.

Thus, the main objective of this network meta-analysis (NMA) was to determine the effects of different physical activity interventions on the exercise capacity, as measured by the 6 MWT, of patients with COPD. In addition, we aimed to determine whether supervised or unsupervised programs could be more effective in improving exercise capacity in patients with COPD.

## 2. Methods

The methodology of this NMA followed the Preferred Reporting Elements for Systematic Reviews that Incorporate Network Meta-Analysis (Supplementary Table S1) [22] and was performed according to the Cochrane Collaboration handbook [13,23]. Additionally, the protocol of this systematic review and NMA was previously registered in the International Prospective Registry of Systematic Reviews (PROSPERO), with the registration number CRD42021228433.

### 2.1. Search Strategy and Selection Criteria

Two independent reviewers (S.P. J and C.A.-B.) conducted a literature search of PubMed, SCOPUS, WoS, Physiotherapy Evidence Database, Cochrane Central Register of Controlled Trials, the Cochrane Central Register of Systematic Reviews, and the Web of Science databases from inception to April 2022.

The aim of the search was to identify published randomized clinical trials (RCTs) on the effect of physical activity programs on exercise capacity, measured as the 6 MWT, of patients with COPD. The search strategy included the following relevant terms combined using Boolean operators: “physical activity”, “pulmonary rehabilitation”, “yoga”, “active mind–body movement therapies”, “aerobic exercise”, “exercise capacity”, “6 MWT”, “COPD”, and “chronic obstructive pulmonary disease”. The full PubMed database search strategy is available in the supplementary material (Supplementary Table S2). Additionally, we checked the reference lists of the included studies and references in previous systematic reviews and meta-analyses for potentially relevant studies.

### 2.2. Eligibility

This NMA included studies on the effectiveness of different types of exercise in improving the exercise capacity, as measured by the 6 MWT, of patients with COPD.

For the selection of studies, the following inclusion criteria were defined: (a) type of studies: RCTs; (b) population: adults with COPD, at any stage of disease; (c) intervention: any type of physical activity intervention defined as repeated bouts of exercise over time aimed at improving physical fitness and involving multiple sessions over several weeks, months, or years of training; (d) outcome: change in exercise capacity, measured using 6 MWT. No language limitation was applied.

Studies were excluded if they included (a) patients suffering from other respiratory pathologies, including COPD, where data from patients with COPD could not be extracted separately; (b) interventions where physical activity was combined with other health interventions; (c) studies with a lack of data to estimate the effect of the interventions. Finally, studies were excluded when they were not RCTs, as well as those RCTs with a lack of data to estimate the effect of the interventions.

### 2.3. Data Extraction

Data from the included RCTs were extracted using an ad hoc form, including information on the author, country, sample size, population characteristics (mean, age, degree of COPD, and FEV1 percentage), intervention characteristics (duration, in weeks or months, frequency, type, timing, and intensity of training regime), type of physical activity performed by the intervention group, and the activity performed by the control group.

Two authors (S.P. J and C.A.-B.) independently conducted the literature search and data extraction. Discrepancies were resolved by a third reviewer who was responsible for resolving any disagreements (I.C.R.).

### 2.4. Categorization of the Interventions

The exercise interventions included in the selected studies were categorized as active mind–body movement therapies (AMBMT), combined training (COMB), endurance (END), pilates, PR, home-based pulmonary rehabilitation (HMPR), and urban training. (i) AMBMT includes interventions (yoga, tai chi, and qigong) supervised and performed as a form of the PR program. These interventions are based on strength and flexibility exercises combined with the patient’s deep and controlled breathing. (ii) COMB includes training programs that combine two interventions in the same session (strength and endurance training). (iii) END refers to aerobic training (i.e., walking and cycling). (iv) Pilates intervention is a supervised pilates training program. (v) PR programs consist of comprehensive programs including different components, such as warm-up, strength exercises, aerobic exercise, breathing, and health education. (vi) Self-monitored HMPR is an alternative to outpatient rehabilitation [24,25] in which patients are instructed in the implementation of the exercise program, which they perform at home. (vii) Urban training includes PR programs that implement a series of urban walking circuits adapted to the characteristics of the patient to encourage daily walking.

In addition, we categorized the interventions into supervised and unsupervised, where supervised referred to those programs implemented in a hospital under the supervision of health professionals; in the case of HMPR, programs that were supervised either by telephone or via home visits by the health professional, including booster sessions.

### 2.5. Assessment of Risk of Bias

The Cochrane Collaboration tool to assess the risk of bias (RoB2) [26] was used to assess the risk of bias of the included studies based on 5 domains: randomization process, deviations from planned interventions, missing outcome data, outcome measurement, and selection of the reported outcome. Overall bias was considered “low risk of bias” if the study was finally classified as “low risk” in all of its domains, “some concerns” if at least one of the domains was rated as “some concerns”, and “high risk of bias” if at least one of its domains had been classified as “high risk” or several domains classified as “some concerns”.

Two investigators (S.P. J and C.A.-B.) independently performed the risk of bias assessment of the included studies. Disagreements were resolved by consensus or discussion with a third reviewer, I.C.R.

### 2.6. Rating of the Quality of the Evidence

To evaluate the quality of the evidence as well as to make recommendations, the Rating of Recommendations, Evaluation, and Development evaluation (GRADE) tool was used [27]. The rating was based on the design of the studies, risk of bias, indirect evidence, inconsistency, publication bias, and imprecision, with each study rated as high, moderate, low, and very low value of evidence.

### 2.7. Data Synthesis and Statistical Analysis

To carry out the analysis, we followed the detailed steps. We started to evaluate transitivity by testing whether the clinical characteristics of the included samples were similar at baseline. We assumed that the populations included in these studies were similar in their initial distribution of potential effect modifiers (age, sex, and severity).

To assess the distribution of the available evidence, we used a network geometry graph, with the size of the nodes representing the number of trials included for each intervention proportionally and the width of the continuous line connecting the nodes corresponding to the number of trials that compare directly the two interventions [28].

Consistency was assessed by checking whether the intervention effects estimated from direct comparisons were consistent with those estimated from indirect comparisons.

Finally, we used the relative rankings of the treatments to establish the ranking among the exercise interventions, which we graphically represented using rankograms. Additionally, we estimated the area under the cumulative ranking (SUCRA) for each intervention, in which each intervention could obtain a score for SUCRA between 0 and 1, 0 being assigned to the worst intervention and 1 to the best one [28,29].

Subsequently, the DerSimonian–Laird random-effects method was used to compute standard paired meta-analyses for direct comparisons between the interventions and the control/no intervention [30]. Furthermore, statistical inconsistency was analyzed using the I^2^ statistic, which was considered not important (I^2^: 0–40%), moderate (I^2^: 30–60%), substantial (I^2^: 50–90%), or considerable (I^2^: 75–100%); likewise, the corresponding *p*-values were considered [24]. Finally, the size and clinical relevance of the heterogeneity were determined using the τ^2^ statistic. An estimate of τ^2^ of 0.04 was interpreted as a low degree of clinical relevance of heterogeneity, 0.14 as moderate, and 0.40 as substantial [31]. A forest plot and classification table were generated to display the results graphically. Direct and indirect comparisons were summarized in an ad hoc table.

To assess the robustness of the estimates and to detect whether any particular study accounted for a large proportion of the heterogeneity, we performed a sensitivity analysis. Finally, Egger’s regression asymmetry test was used to assess publication bias. For this purpose, the statistical software StataSE, version 16.0 (StataCorp, College Station, TX, USA), was used.

Following similar procedures, we conducted a second NMA to determine whether the effectiveness of supervised and unsupervised PR and HMPR programs on the exercise capacity of patients with COPD was affected by the many differences between them.

For all of the analyses, in cases where more than one study provided data referring to the same sample, the study reporting the most detailed data or with the largest sample size was included. Following the Cochrane Collaboration Handbook recommendations, when data on the standard deviation (SD) of change in the functional capacity from baseline were missing, estimates were based on standard error (SEs), 95% confidence intervals (CIs), *p*-values, or t-statistics to calculate the SD.

## 3. Results

**Figure 1:**
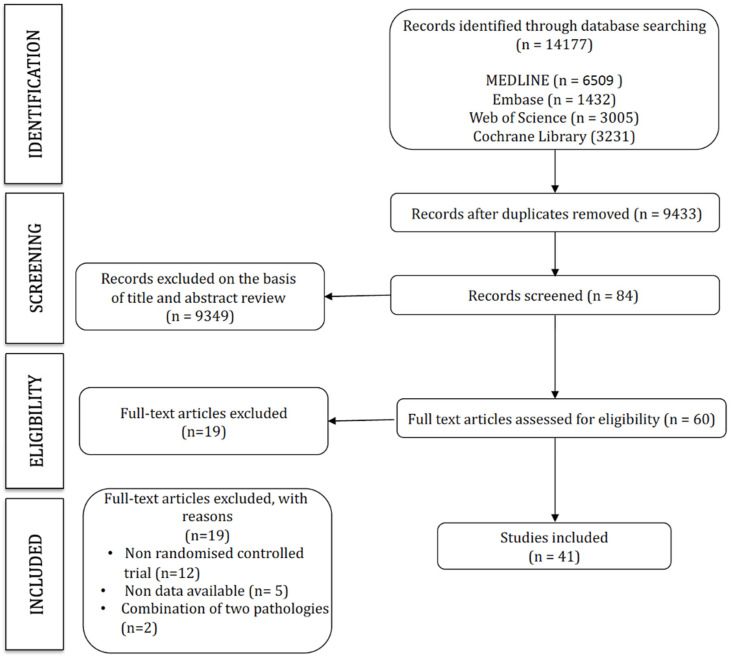
PRISMA flow diagram.

The search strategy retrieved 14,177 studies, of which 41 were included in this network meta-analysis (Figure 1). The characteristics of the included studies are displayed in Table 1, and the characteristics of the interventions are included in Table 2 (Figure 1).

### 3.1. Risk of Bias

Thirteen studies were assessed as having a low risk of bias, two as having some concerns, and twenty-six as having a high risk of bias. Considering each specific domain, the randomization process domain was scored as low risk in 68.3% of the studies and as some concern in 29.3%. The deviations from the intended interventions domain were scored as low risk in 90.2% of studies and as some concern in 9.8%. The missing outcome data domain was scored as low risk in 80.5% of studies and as high risk in 19.5%. The measurement of the outcome domain was scored as a high risk of bias in 53.7% of studies and as low risk in 46.3%. The selection of the reported result domain was scored as some concerns in 12.2% of studies and as low risk in 87.8% (Supplementary Figures S1 and S2).

Additionally, the GRADE results are available in Supplementary Table S3.

### 3.2. Network Analyses

After the evaluation of transitivity, the transitivity assumption was met for all comparisons, including participants with similar baseline characteristics (gender, age, and disease severity) (Supplemental Table S4).

The network diagrams showed the relative amount of evidence available for exercise interventions for exercise capacity measured by the 6 MWT among patients with COPD. The NMA involved 18 direct comparisons for exercise capacity. Most interventions had at least one direct comparison with a control.

### 3.3. Modalities of Exercise and Their Effect on Exercise Capacity

**Figure 2:**
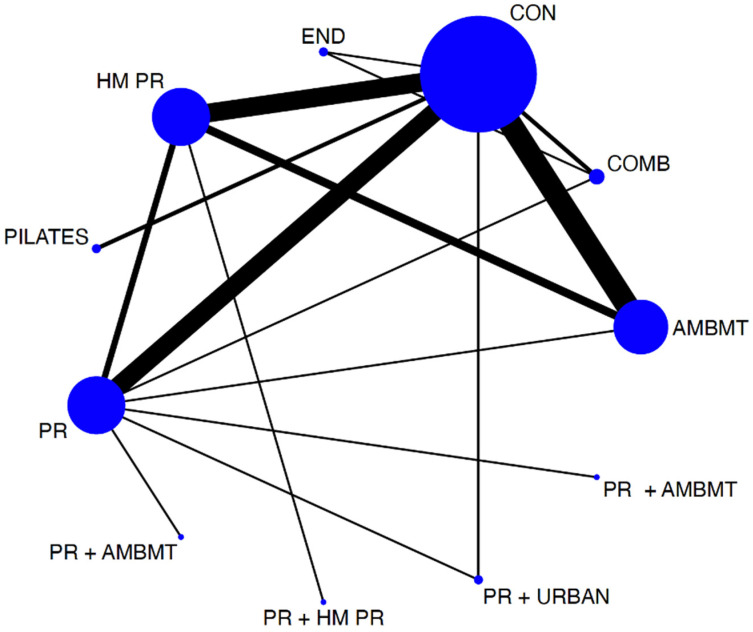
Network of available comparisons between different physical interventions, such as pulmonary rehabilitation programs on exercise capacity. Size of node is proportional to number of trial participants, and thickness of continuous line connecting nodes is proportional to number of participants randomized in trials directly comparing the two treatments. AMBMT: active mind–body movement therapies; COMB: combined; CON: control. HMPR: home pulmonary rehabilitation program; PR: pulmonary rehabilitation program; END: endurance; PR + AMBMT: pulmonary rehabilitation program + active mind–body movement therapies; PR + HMPR: pulmonary rehabilitation program + home pulmonary rehabilitation program; PR + URBAN: pulmonary rehabilitation program + urban training.

Table 3 shows the ES estimates for exercise capacity scores using the 6 MWT test. The highest effects for pairwise comparisons were for PR+ URBAN versus the control group (ES, 1.50; 95% CI 0.46 and 2.55). In addition, Pilates (ES, 1.32; 95% CI, 0.18 to 2.45) versus the control group and AMBMT (ES, 0.96; 95% CI, 0.61 to 1.31) and PR interventions (ES, 0.91; 95% CI, 0.52 to 1.31) versus the control group showed positive effects on exercise capacity measured with the 6 MWT. This was followed by COMB (ES, 0.90; 95% CI 0.07 to 1.74) interventions versus the control group (Figure 2 and Table 3).

When analyses were limited to supervised and unsupervised PR and HMPR programs, the largest effects were found for supervised PR (ES, 0.85 CI 0.46 to 1.23) and supervised HMPR (ES, 0.84 CI 0.33 to 1.36) versus the control group (Supplementary Table S5 and Supplementary Figure S3).

### 3.4. Best Treatment Probabilities

For the 6 MWT exercise capacity score, the highest SUCRA was observed for PR + URBAN (82.5%) and pilates (73.6%) (Supplementary Tables S6 and S7 and Supplementary Figures S4 and S5).

### 3.5. Sensitivity Analysis, Heterogeneity, and Publication Bias

AMBMT vs. HMPR showed considerable heterogeneity for exercise capacity (I^2^ = 89.6, τ^2^ = 0.3080). Furthermore, AMBMT vs. control showed substantial heterogeneity (I^2^ = 78.8, τ^2^ = 0.223). Direct comparisons for HMPR and PR vs. control showed substantial heterogeneity (I^2^: 75.4, τ^2^ = 0.1903; I^2^: 71.2, τ^2^: 0.2044, respectively). All other direct comparisons showed no significant heterogeneity (*p* > 0.05) (Supplementary Tables S8 and S9).

Finally, publication bias was found in the AMBMT vs. control (*p* = 0.526) and combined vs. control (*p* = 0.441) tests for exercise capacity (Supplementary Table S10). The funnel plots are shown in Supplementary Figure S6.

## 4. Discussion

Although numerous studies have reported on the effectiveness of different exercise interventions in improving exercise capacity in COPD patients, as measured by the 6 MWT test, an up-to-date comprehensive review comparing the effect of each exercise modality is lacking. This NMA, which includes 41 RCTs with 3155 participants, provides evidence that exercise is an effective therapeutic strategy for exercise capacity among patients with COPD. In addition, analysis of the available evidence indicates that the best exercise interventions to improve exercise capacity as measured by the 6 MWT are PR programs implemented with urban training, followed by Pilates and AMBMT. In addition, our data show that these interventions also obtained the best benefits when supervised.

The largest effects of this NMA were found for PR, which is a comprehensive intervention that has been shown to improve dyspnoea, exercise capacity, and health-related quality of life in patients with COPD [73]. The Global Initiative for the management of COPD (GOLD) includes among the benefits of these programmes the reduction of symptoms, complications, and exacerbations, as well as improved exercise tolerance, better health, and a reduction in mortality [74]. Recent evidence-based clinical practice guidelines and studies have concluded that PR is widely accepted as the most effective nonpharmacological therapy in the treatment of COPD [75], but poor access to PR programs hampers the widespread implementation of this effective intervention. For this reason, different authors have proposed the use of self-controlled HMPR as an opportunity to offer different PR settings tailored to individual needs with the aim of improving patient access to this intervention [36].

Pilates is a method of therapeutic exercise in which breathing, including full inhalation and exhalation and posterior lateral breathing [76,77], are key components [78]. The principles of the pilates exercises emphasize improved breathing, concentration, motor control, axial elongation, and flexibility, all of which are associated with increased strength [79]. In addition, breathing training emphasizes the lateral expansion of the rib cage by maintaining constant activation of the deep abdominal muscles, with active contraction of the transversus abdominis and pelvic floor muscles during inhalation and exhalation [80]. The pilates program interventions are designed to improve muscular strength and endurance, flexibility, posture, and balance and are easy to start and maintain [81].

Patients in the AMBMT [56,59,60,82] interventions received guided and supervised tai chi, qigong, or yoga sessions (with different movement routines) [83,84], as well as audiovisual material with an exercise program to be performed at home. Participants were asked to coordinate their deep breathing with the prescribed movements. Tai chi is a light to moderate aerobic activity and includes strength training of the lower and upper limb muscles without resistance [85]. The benefits of a supervised program, implemented with the performance of an exercise program at home, as well as daily physical activity to increase adherence to treatment, could improve the effectiveness of this exercise.

The abovementioned interventions, which have been found to have the greatest significant improvement, were supervised by physiotherapists or specialized therapists, a factor to be considered when analyzing our results. Research has shown that weekly telephone contact and supervised rehabilitation sessions, both weekly and monthly, during HMPR programs had modest effects on maintaining improvements in exercise tolerance and general health throughout the intervention [86]. Face-to-face program supervision or booster sessions in the PR or HMPR programs leads to an increase in adherence, perhaps the most important problem encountered in chronic disease interventions.

Exercise capacity has been the focus of numerous studies on COPD in recent years. PR and exercise interventions have shown a greater effect than pharmacological treatments in improving the symptoms of patients with COPD. Similar to our results, previous studies have shown that people with COPD can expect an increase in exercise capacity, as measured by 6MWD, through exercise interventions, including PR. However, future studies should consider additional factors, such as the height of the subject, which can potentially influence the distance covered and the effectiveness of ambulation. In addition, the body weight of the patient directly affects the work/energy required to perform the gait, which could directly affect the distance walked by the patient, as energy expenditure is determined as force and distance travelled. Therefore, it seems logical to include strength (body weight) as well as distance walked when assessing an individual’s walking capacity [12].

Several reasons could explain the effectiveness of exercise interventions on exercise function in patients with COPD. Previous research has associated decreased peripheral muscle strength (measurements of the isometric strength of the quadriceps, handgrip, and respiratory muscles) with reductions in ventilatory capacity, which independently contribute to reductions in exercise capacity [87]. Peripheral muscle weakness has been shown to be a contributing factor to exercise limitations in COPD [88,89]. Functional and morphological abnormalities of skeletal muscle have been found in patients with COPD, probably related to factors such as hypoxemia, malnutrition, underuse, and aging, leading to the physical deconditioning of these patients [90,91,92,93]. An increase in quadriceps femoris strength after training may reduce the sensation of muscle fatigue, a limiting factor in exercise for COPD patients [32,89,94,95]. In studies of healthy elderly subjects, resistance training was associated with modest but significant increases in the oxidative capacity of skeletal muscle [92], a factor related to the lack of exercise tolerance in patients [92] with COPD. In this regard, it has been suggested that a positive effect of exercise is to improve functional capacity and muscle strength in patients with COPD, reducing their dyspnoea and promoting adherence to exercise programs.

## 5. Study Limitations

This study has some limitations that must be acknowledged. First, it should be noted that we could not consider the intensity of the exercises analyzed because this information was missing in most of the studies. Second, this study cannot provide recommendations on the appropriateness of each type of exercise based on the duration, severity, and progression of the disease due to the heterogeneity among the studies reviewed. Third, the overall moderate risk of bias in the included studies was mainly due to the lack of information on the bias of the intended interventions (usually related to difficulties in blinding participants, as patients were aware of the intervention). Fourth, the findings should be considered with caution due to the limited number of studies for some interventions, including some of the most effective ones, such as pilates and PR + URBAN. Fifth, within the pulmonary rehabilitation intervention, not all studies follow the quality standards according to the modern concept of pulmonary rehabilitation programs. Sixth, we could not determine the long-term effects of the interventions or adherence to the programs after the completion of the studies.

## 6. Conclusions

In summary, PR programs implemented with urban circuit training seem to be the most effective type of intervention for improving exercise capacity as measured with the 6 MWT. In addition, pilates interventions have shown similar effects on exercise capacity when both interventions are supervised by physiotherapists. Comprehensive PR programs, preferably implemented within urban exercise training programs, should be considered a low-cost therapeutic strategy to improve exercise capacity in patients with COPD, improving their functional capacity. Our results are based on evidence from RCTs and represent a comprehensive research effort aiming to synthesize the best available evidence on the type of exercise intervention that is most effective in improving exercise capacity in these patients. However, more studies are needed to provide evidence on the effectiveness of exercise by disease severity as well as exercise intensity to improve the effectiveness of exercise recommendations for increasing the exercise capacity of COPD patients.

## Figures and Tables

**Table 1:** Characteristics of the population described in the included studies.

Study Characteristics			Population Characteristics					
Study	Country	N	Age (y), Mean ± SD	Disease Severity	FEV1 % Pred	N Cases Failed Program	Groups by Intervention	N Intervention
**Simpson et al., 1992** [32]	Canada	34	73 ± 4.8 70 ± 5.7	Severe COPD	39.5 ±18.96 39.2 ± 21.39	3 3	**IG:** COMB **CG:** CON	14 14
**Bernard et al., 1999** [33]	Canada	45	64 ± 7 67 ± 9	Moderate to Severe	45 ± 15 39 ± 12	5 4	**IG:** COMB **CG:** PR	21 15
**Spencer et al., 2010** [34]	Australia	48	65 ± 8 67 ± 7	COPD Moderate COPD	57 ± 21 60 ± 16		**IG:** PR+ HMPR **CG:** HMPR	24 24
**Nguyen et al., 2013** [35]	EEUU	125	68.5 ± 11.0 68.2 ± 9.9 69.3 ± 8.0	Moderate to severe COPD	53.3 ± 20.4 50.6 ± 18.2 49.4 ± 19.8		**IG:** HMPR **IG:** HMPR **CG:** CON	43 41 41
**Maltais et al., 2008** [36]	Canada	252	66 ± 9 66 ± 9	Severe COPD	43± 13 46 ± 13		**IG:** PR **CG:** HMPR	126 126
**Oh, 2003** [37]	Korea	23	64.8 ± 7.84 66.8 ± 12.29	Moderate to severe COPD	42.12 ± 15.07 44.91 ± 17.75		**IG:** HMPR **CG:** CON	15 8
**Donesky-Cuenco et al., 2009** [38]	EEUU	29	72.2 ± 6.5 67.7 ± 11.5	Moderate COPD	72.2 ± 6.5 67.7 ± 11.5		**IG:** AMBMT **CG:** CON	14 15
**Liu et al., 2012** [39]	China	140	61.82 ± 7.69 61.34 ± 8.34 62.2 ± 6.34	Moderate COPD	74.43 ± 12.93 75.31 ± 12.84 75.31 ± 13.79		**IG:** AMBMT **IG:** PR **CG:** CON	51 32 35
**Yeh et al., 2010** [40]	EEUU	10	65 ± 6 66 ± 6	Moderate to severe COPD	53 ± 7 47 ± 7		**IG:** AMBMT **CG:** CON	5 5
**Niu et al., 2014** [41]	China	40	59.7 ± 2.76 61.3 ± 2.89	Severe COPD	41.9 ±5.50 43.7 ± 5.16		**IG:** AMBMT **CG:** CON	20 20
**Gu et al., 2012** [42]	China	66	67 ± 8 69 ± 9	Moderate to severe COPD	48.4 ± 18.7 51.6 ± 20.0	2 5	**IG:** AMBMT **CG:** CON	33 30
**Ng et al., 2014** [43]	China	192	74.13 ± 6.81 74.16 ± 6.46	Moderate to severe COPD	64.1± 22.12 56.4 ± 23.81		**CG:** PR **IG:** PR+AMBMT	98 94
**Chan et al., 2011** [44]	China	206	71.7 ± 8.2 73.6 ± 7.5 73.6 ± 7.4	Mild to severe COPD	50.1 ± 21.8 56.4 ± 25.6 55.1 ± 23.3		**IG:** AMBMT **IG:** HMPR **CG:** CON	70 69 67
**Gottlieb et al., 2011** [45]	Denmark	42	74.1 ± 66–82 73.2 ± 67–88	Moderate COPD	64.27 ± 7.9 67.05 ± 8.8		**IG:** PR **CG:** CON	22 20
**Román et al., 2013** [46]	Spain	97	64.9 ± 62.1–67.7 64.1 ± 59.9–68.2 63.4 ± 60.4–66.4	Moderate COPD	60.9 ± 56.3–65.5 59.9 ± 54.9–64.8 60.1± 55.6–64.4	6 11 9	**IG:** PR **IG:** PR **CG:** CON	26 22 23
**Engstrom et al., 1999** [47]	Sweden	55 (50)	66.0 ± 5.4 66.8 ± 5.4	Severe COPD	30.7 ± 11.4 34.1± 10.2	2 3	**IG:** PR **CG:** CON	26 24
**Singh et al., 2003** [48]	India	40	59.37 ± 6.4 59.37 ± 6.4	Severe COPD	28± 7.5 26 ± 7.1		**IG:** PR **CG:** CON	20 20
**Borghi-Silva et al., 2009** [49]	EEUU	34	67 ± 10 67 ± 10	Severe COPD	33± 9 35 ± 11		**IG:** PR **CG:** CON	20 14
**Muñoz Fernández et al., 2009** [50]	Spain	41	66 ± 8 70 ± 5	Severe COPD	33 ± 10 38 ± 12		**IG:** HMPR **CG:** CON	27 14
**Theander et al., 2009** [51]	Sweden	26	66 ± 6 64 ± 6	Severe COPD	35.1 ± 7.6 32.3 ± 9.5		**IG:** PR **CG:** CON	12 14
**Ghanem et al., 2010** [52]	Egypt	39	56.96 ± 11.59 56.43 ± 9.03	Moderate to severe COPD	29.44 ± 13.14 23.21 ± 7.70		**IG:** HMPR **CG:** CON	25 14
**Pleguezuelos et al., 2013** [53]	Spain	125	70.2 ± 2.5 70.5 ± 2.5 72.4 ± 1.7	Severe or very severe COPD	32.0 ± 1.2 31.8 ± 1.0 31.6 ± 0.8		**IG:** PR+ URBAN **IG:** PR **CG:** CON	34 37 54
**De Sousa Pinto et al., 2014** [54]	Spain	50 (41)	68.9 ± 9.2 71.9 ± 7.6	Severe or very severe COPD	33.5 ± 7.3 34.5 ± 9.5	6 3	**IG:** HMPR **CG:** CON	23 18
**Chan et al. 1., 2013** [55]	China	206	71.7 ± 8.2 73.6 ± 7.5 73.6 ± 7.4	Mild to severe COPD	50.1 ± 21.8 56.4 ± 25.6 55.1 ± 23.3		**IG:** AMBMT **IG:** HMPR **CG:** CON	70 69 67
**Zhang et al., 2016** [56]	China	130	64.77 ± 11.07 63.34 ± 7.86 62.35 ± 9.27		59.12 ± 4.13 57.39 ± 5.37 58.11 ± 4.37		**IG:** AMBMT **IG:** HMPR **CG:** CON	42 43 45
**Ranjita et al., 2016** [57]	India	72	53.69 ± 5.66 54.41 ± 5.40	Moderate COPD	47.2–52.8 41.7–58.3	5 4	**IG:** AMBMT **CG:** CON	36 36
**Gupta et al., 2014** [58]	India	50	52.5 ± 3.9 52 ± 4.1	Moderate to severe COPD	51.1 ± 8.7 49.6 ± 8.6		**IG:** AMBMT **CG:** CON	25 25
**Xiao et al., 2015** [59]	China	126	72.2 ± 1.7 70.9 ± 1.4	Moderate to severe COPD	41.5 ± 4.5 40.7 ± 4.0		**IG:** AMBMT **IG:** HMPR	63 63
**Ng et al., 2011** [60]	China	80	71.75 ± 1.05 73.12 ± 1.33	Severe COPD	37.13 ± 2.22 36.75 ± 2.11		**IG:** AMBMT **CG:** CON	40 40
**Papp et al., 2017** [61]	Sweden	36	61 ± 40–76 69 ± 43–84	Mild to severe COPD	67.6 ± 20.4 64.3 ± 15.4		**IG:** AMBMT **IG:** COMB	19 17
**Daabis et al., 2016** [62]	Egypt	45	58.7 ± 7 61 ± 8 60 ± 8	Moderate COPD	56.4 ± 8.3 53.2 ± 9.5 54.6 ± 7.1		**IG:** COMB **IG:** END **CG:** CON	15 15 15
**Fukuoka et al., 2016** [63]	China	8	74.6 ± 6.7 77.0 ± 7.0	Moderate COPD			**IG:** PR+ AMBMT **IG:** PR	5 3
**Hansen et al., 2019** [64]	Denmark	134	68.4 ± 8.7 68.2 ± 9.4	Severe COPD	32.6 ± 10.3 33.7 ± 8.4		**IG:** HMPR **IG:** PR	67 67
**Hagag et al., 2019** [65]	Egypt	38	43.05 ± 2.07 42.09 ± 3.08	Moderate COPD	62.72 ± 3.34 63.59 ± 3.61		**IG:** PILATES **CG:** CON	19 19
**Wen et al., 2020** [66]	China	60	63.74 ± 3.24 64.10 ± 3.56				**IG:** PR **CG:** CON	30 30
**Kilic et al., 2021** [67]	Turkey	58	68.4 ± 8.91 69.87 ± 10.46	Mild to very severe COPD	50.26 ± 19.08 57.52 ± 19.72		**IG:** PR **IG:** HMPR	27 31
**Kantatong et al., 2019** [68]	Thailand	50	69.68 ± 7.76 67.48 ± 10.17	Mild to moderate COPD	68.21 ± 21.63 68.37 ± 18.90		**IG:** AMBMT **CG:** CON	25 25
**Babkina et al., 2017** [69]	Ukraine	102	69.3 ± 5.6 67.2 ± 6.1				**IG:** AMBMT **CG:** CON	48 54
**Pradella et al., 2015** [70]	Brazil	29	62.4 ± 10.7 65.3 ± 8	Mild to very severe COPD	62.4 ± 10.7 54 ± 26.2	3 3	**IG:** HMPR **CG:** CON	29 15
**Kraemer et al., 2021** [71]	EEUU	92	68.6 ± 9.2 67.5 ± 7.7	Moderate to severe COPD	57.8 ± 14.3 59.6 ± 14.8		**IG:** AMBMT **CG:** CON	61 31
**Barakat et al., 2008** [72]	France	80	63.7 ± 11.9 65.9 ± 10.3	Severe COPD	41.9 ± 2.6 43.33 ± 3.6		**IG:** PR **CG:** CON	40 40

**IG:** Intervention group; **CG:** control group; **COPD:** chronic obstructive pulmonary disease.

**Table 2:** Characteristics of the interventions described in the included studies.

Study	Groups by Intervention	Intervention	Time (min)/rep	Intensity	Duration (wk)	Frequency (x/wk)
Simpson et al., 1992 [32]	IG: COMB	5 min warm up of low resistance CYC + 2 min of low resistance arm exercise on AC + WLE single arm curl, single leg extension, and single leg press exercise. Normal breathing during the lifting phase of the exercise.	3 series of 10 rep	50% (1 week)–85% 1RM final	8	3
	CG: CON	Usual medical care.				
Bernard et al., 1999 [33]	IG: COMB	Aerobic training + WLE: pectoralis major, elbow flexion, shoulder adduction (latissimus dorsi), leg press, and bilateral knee extension.	30 min aerobic + 45 ST 2 series of 8/10 rep. Thereafter, it increased by more than 10 rep	Work rate 80% of peak work in incremental exercise test + 60% 1RM	12	3
	CG: PR	Leg exercise on ergocycle + relaxation and breathing exercises.	30 min + 45 relax and breathing	Work rate 80% of peak work in incremental exercise test	12	3
Spencer et al., 2010 [34]	IG: PR+ HMPR	1 day gym: Walk + SC + AC + LWM + AWM + UAE/HMPR: Walk + S, Sq, S/S, UAE.	1 day gym: 20 min W + 2 min SC + 30 min AC, LWM, AWM, UAE. Total 70 min/HM PR: 30 min W + 30 min S, SQ, S/S, UAE. Total 60 min		8	1 day gym + 4 HMPR
	CG: HMPR	HMPR: Walk + S, Sq, S/S, UAE.	HM PR: 30 min W + 30 min S, SQ, S/S, UAE. Total 60 min			5 HMPR
Nguyen et al., 2013 [35]	IG: HMPR	Education and energy saving strategies + END (walk, CYC, swimming) + arm ST (biceps, triceps, side arm raises, and upper arm raises) program + self-monitoring of exercise and respiratory symptoms. Reinforcement emails, interactive web modules, live groups, and chat sessions.	END: 30 min + dyspnea and exercise consultation (1–1.5 h) + structured education (six 1 h sessions)		12 months	END: 4/arm strengthening: 3
	IG: HMPR	Education and energy saving strategies + END (walk, CYC, swimming) + arm ST (biceps, triceps, side arm raises, and upper arm raises) program + self-monitoring of exercise and respiratory symptoms. Reinforcement telephone calls (5–10 min), paper modules, and face-to-face groups sessions.	END: 30 min + dyspnea and exercise consultation (1–1.5 h) + structured education (six 1 h sessions)			END: 4/arm strengthening: 3
	CG: CON	Monthly face-to-face education classes (nutrition and general safety with medications). Weekly phone calls with health information.				1
Maltais et al., 2008 [36]	IG: PR	END (CYC) + ST exercises.	END: 25–30 min + ST: 30 min, starting with 1 set of 10 rep e/e. Max 3 sets. Increased resistance (elastic bands, sandbags, and weight against gravity)	80% peak work capacity during incremental exercise	3 months (8 weeks (3 times a week) + 4 weeks education program)	3
	CG: HMPR	END (CYC) + ST exercises + telephone calls to reinforce.	END: 40 min + ST: 30 min, starting with 1 set of 10 rep e/e. Max 3 sets. Increased resistance (elastic bands, sandbags, weight against gravity)	60% max work rate achieved during a test of peak exercise capacity		3
Oh., 2003 [37]	IG: HMPR	Education + IMT + exercise training (stretching, walk, stair climbing, and ST of upper and lower limbs with a theraband) + psychosocial component (relaxation and telephone calls).	5 min inspiration training each time (five times/day) + stretching 5 times/day (10 times each time) + relaxation technique (2/day)		8	Telephone visits: 2
	CG: CON	Educational advice.				
Donesky-Cuenco et al., 2009 [38]	IG: AMBMT	Yoga asanas (stretching movements during exhalation) with breathing pranayama techniques + videotape of one yoga class to practice daily at home.	1 h yoga sessions		12	2 supervised + 7 at home
	CG: CON	Educational advice and were offered the yoga program at the conclusion of the 12 weeks period.				
Liu et al., 2012 [39]	IG: AMBMT	Health qigong training (selected routines).	1 h		6 months	3
	IG: PR	END (walk, CYC) + pursed-lip breathing + were encouraged to participate in peer-led weekly walking and ball game activities.	1 h			3
	CG: CON	Health education and were advised to continue exercising by themselves.				
Yeh et al., 2010 [40]	IG: AMBMT	Warm-up exercises (WLE+ arm swinging + gentle stretches of the neck, shoulders, spine, arms, and legs, visualization techniques, and traditional breathing methods) + 5 tai chi movements + 35 min instructional videotape with exercises to practice at home at least 3 times/week.	1 h		12	2 + 3 at home
	CG: CON	Usual medical care.				
Niu et al., 2014 [41]	IG: AMBMT	Warm-up + tai chi program + post-exercise relaxation + DVD developed for the home sessions.	10 min warm up + 30 min tai chi + 10 min relaxation	Intensity was adjusted for each COPD patient according to her/his toleration of the program	6 months	4 + 3 at home
	CG: CON	Usual medical care + lip breathing + walking daily.	10–15 min lip breathing + 30 min walking daily			
Gu et al., 2012 [42]	IG: AMBMT	Educational sessions + simplified 24-style Taijiquan routines and their breathing method + weekly diary of physical activity measured with pedometers.	60 min		3 months	3
	CG: CON	Educational sessions + telephone monitoring once a month.	2 h			
Ng et al., 2014 [43]	CG: PR	Warm-up + cooldown exercise + 2 END activities (TMW + lower limb ergometry exercise) + relaxation + HM PR exercises (warm-up + theraband exercises + aerobic exercises + cooldown + relaxation).	5 min warm-up + 20 min each END exercise + 15 min rest between e/e + 5 min cooldown + 15 min relaxation + 1 h daily of unsupervised home exercises (5 min warm-up + 5 min of theraband exercises+ 30 min of aerobic exercises + 5 min cooldown + 15 min relaxation)		6	2 (6 weeks) + after 5–7 at home
	IG: PR+ AMBMT	Warm-up + cooldown exercise + 2 END activities (TMW+ lower limb ergometry exercise) + tai chi (5 forms of Sun Style) + HM PR exercises (warm-up + theraband exercises + aerobic exercises + cooldown + tai chi).	5 min warm-up + 20 min each END exercise + 15 min rest between e/e + 5 min cooldown + 15 min tai chi + 1 h daily of unsupervised home exercises (5 min warm-up + 5 min of theraband exercises + 30 min of aerobic exercises + 5 min cooldown + 15 min tai chi)			
Chan et al., 2011 [44]	IG: AMBMT	TCQ movements emphasized the elements of breathing + DVD to facilitate daily self-practice + diary for recording the frequency.	60 min		3-month	2 + daily at home
	IG: HMPR	PLB + DB + self-paced walking + diary for recording the frequency.				daily at home
	CG: CON	Were advised to maintain routine activities.				
Gottlieb et al., 2011 [45]	IG: PR	Preliminary motivational personal interview + intensive physical training and educational phase supervised + final interview follows up. Training (END + static circuit training + walk + breathing techniques). Smoking cessation counseling + dietary intervention.	Physical training: 90 min sessions	Intensity of 16–17 Borg scale (20 point)	7 + 6 months maintenance phase	Physical training: 2/Educational sessions: 1
	CG: CON	Standard community care.				
Román et al., 2013 [46]	IG: PR with maintenance group	Education program + respiratory physiotherapy (breathing + DB + exercises for the chest wall and abdominal muscle walls) + peripheral muscle training (abdominal and upper and lower limb exercises, shoulder, and full arm circling, WLE) + maintenance program.	60 min (respiratory physiotherapy 15 min + 45 min exercises training. 8–10 rep e/e) + 45 min education session (weeks 1, 6 and 12)	Low-intensity peripheral muscle training	3 months + 9 months maintenance phase	3 + 1 (maintenance program)
	IG: PR without maintenance group	Education program+ respiratory physiotherapy (breathing + DB + exercises for the chest wall and abdominal muscle walls) + peripheral muscle training (abdominal and upper and lower limb exercises, shoulder, and full arm circling, WLE) + routine care after completing the 3 months PR.			3 months	3
	CG: CON	Continue their routine care.			3 months	
Engstrom et al., 1999 [47]	IG: PR with maintenance group	CYC + arm training + training in breathing techniques (PLB+ DB).	45 min (first 15 min breathing techniques) + 30 min CYC +30 min HMPR daily: thorax and shoulder-girdle training + muscle ST (theraband) + energy saving techniques (2 sessions) + nutrition advise + educational sessions (2)	42–85% W max at 2 min intervals after 5 min warming up at 50% of W max. These 2 min intervals (42–85%) were repeated for 25 min at most. Borg score of 15 (hard) for “effort” + 30 min HMPR	12 months	2 (6 weeks) + 1 (6 weeks) + once every second week (6 weeks) + 1 a month for the remaining period + HMPR daily
	CG: CON	Usual outpatient care.				
Singh et al., 2003 [48]	IG: PR	Breathing (PLB + DB + controlled coughing + postural bronchial drainage) + lower extremity exercise (walk + PLB) + energy conservation techniques.	30 min twice a day	Walk submaximal speed (twice a day) + PR 30 min (twice a day)	4	twice daily
	CG: CON					
Borghi-Silva et al., 2009 [49]	IG: PR	Aerobic training (ST lower and upper limbs + TMW) + stretching exercises + DB (hamstrings, quadriceps, calves, shoulders, neck, and lower back).	30 min aerobic + 10 min stretching	Training intensity in TMW: 70% max speed achieved during the exercise test	6	3
	CG: CON	Vibration and clapping associated with postural drainage for 10 min with supported cough.	10 min			
Muñoz Fernández et al., 2009 [50]	IG: HMPR	Respiratory reeducation + IMT + ST upper limbs (initially isotonic exercises without weights + after WLE) + ST lower limbs (initially isotonic exercises after with resistance + walk).	1 h (15 min respiratory reeducation) + 15 min IMT + 30 min muscular training of upper limbs + 30 min walk	IMT (70% PIM) + Walk (90% velocity reached in the 6 MWT)	12 months	5
	CG: CON	3 respiratory education sessions.				
Theander et al., 2009 [51]	IG: PR	CYC + ST (biceps, latissimus dorsi, and quadriceps) stand-ups, toe raises, step-ups, pelvic-lifts, and sit-ups + after 1 month intervention included home program (daily walk +ST-theraband+ diet education + energy saving techniques + educational advice).	1 h (15 min CYC + ST 10 exercises in 3 rounds per occasion)		12	2
	CG: CON					
Ghanem et al., 2010 [52]	IG: HMPR	Health education + exercise training (PLB + DB) + END (CYC + Walk) + ST (upper extremity. Six to 10 upper-body and lower-body ST exercises) + stretching (hamstrings, quadriceps, calves, shoulders, neck, and lower back).			2 months	every other day
	CG: CON	Usual care.				
Pleguezuelos et al., 2013 [53]	IG: PR+ URBAN	Warm-up exercises (upper and lower limbs and spine) + END (CYC) + ST (upper limbs muscles) + stretching exercises + relax + urban walking circuits.	10 min warm-up+ 25 min CYC + 15 min ST + 10 min stretching+ 10 relax + PR URBAN	Moderate intensity Initial intensity of training 50 W, increasing progressively with tolerance	12	3
	IG: PR	Warm-up exercises (upper and lower limbs and spine) + END (CYC) + ST (upper limbs muscles) + stretching exercises + relax.	10 min warm-up+ 25 min CYC + 15 min ST + 10 min stretching+ 10 relax	Moderate intensity Initial intensity of training 50 W, increasing progressively with tolerance		
	CG: CON					
De Sousa Pinto et al., 2014 [54]	IG: HMPR	Breathing and stretching exercises + ST (upper and lower limbs) + END (walk, stair climbing, CYC, and TMW) + WLE.	Initially, 20–30 min, increased to 1 h. Upper and lower limbs exercises (2 sets of 10 reps e/e without weights to 2 sets of 10 reps with 2 kg weight max for both hands and legs). 3–5 min stair climbing, From 5–10 min to 30 min TMW or CYC. Patients without treadmills or bicycles, walked daily for at least 30 min	Training intensity was adjusted according to the level of dyspnea, dizziness, or leg discomfort	3 months	First 2 weeks (2 visits per week), after twice a month and weekly telephone)
	CG: CON					
Chan et al., 2013 [55]	IG: AMBMT	TCQ movements emphasized the elements of breathing supervised + DVD to facilitate daily self-practice + diary for recording the frequency.	60 min	Were allowed to adjust the position of their extremities and the exercise intensity according to their physical condition	3-month	2+ daily at home
	IG: HMPR	PLB+DB+self-paced walking +diary for recording the frequency.				daily at home
	CG: CON					
Zhang et al., 2016 [56]	IG: AMBMT	QYJJ supervised coordinate breathing with movements + DVD + pictures to facilitate individual practice + group meeting once a week to share experiences.	60 min		6 months	3 group practise + 4 individual practice
	IG: HMPR	Self-pace Walk + DB + PLB + keep a diary to record the frequency.	60 min			7
	CG: CON	Were advised to maintain their routine activities without any extra recommended exercise.				
Ranjita et al., 2016 [57]	IG: AMBMT	Yoga asanas supervised + pranayamas (breathing practices). Deeply relax different muscle groups + slow breathing practices + strengthen respiratory muscles + calm mind + balance emotions + develop internal awareness.	90 min		12	6
	CG: CON	Were offered the 12 weeks yoga program after the intervention period and post-testing were complete.				
Gupta et al., 2014 [58]	IG: AMBMT	Yoga asanas supervised (4 easy asanas) pranayam + usual medical care.	30 min pranayam (5–7 min each asana) (2 x/d)	Low-intensity max heart ranges from 43% to 49% of predicted max	3 months	7 (twice per day)
	CG: CON	Usual medical care.				
Xiao et al., 2015 [59]	IG: AMBMT	LQG training supervised sessions (six distinct movement routines) coordinate their breathing + audiovisual material + advised to walk daily.	45 min. 6 routines/six times each routine. 12–15 min + 30 min walk daily	Low-intensity CYC and ST. ST. Intensity 70% 1RM + CYC (12–14 on Borg 20-scale- 50–60 rpm)	6 months	4
	IG: HMPR	PLB + coordinated breathing + walking exercise program + advised to walk daily.	45 min training + 30 min walk daily			4
Ng et al., 2011 [60]	IG: AMBMT	HQG sessions supervised + package audiovisual materials (8 distint routines).	45 min. 8 distinct movement routines repeated six times. 12–15 min		6 months	4
	CG: CON	Breathing (PLB + coordinated breathing) + walk. Advised to keep daily walking for not less than 30 min.	45 min			4
Papp et al., 2017 [61]	IG: AMBMT	Hatha yoga program supervised + DVD and paper + physical activity after the intervention.	60–70 min	75% of the results of 6 MWT + low-intensity resistance training with free weights.	12	2
	IG: COMB	CYC + ST training (with gym equipment and stationary exercise bikes).	60–70 min. ST: 2–4 sets of 10–20 rep e/e. Total 10–12 exercises+ 10–15 min CYC	Workload at 50–80% 1RM. 1RM repeated every 2 weeks to re-establish the workload		2
Daabis et al., 2016 [62]	IG: COMB	TMW + WLE	60 min: 30 min TMW+ 30 min WLE + pacing for breathing		8	3
	IG: END	ST (weight training machines (pectoralis major, deltoids, biceps brachii, triceps and quadriceps muscles)).	60 min: 30 min ST. 3 sets of 12 reps with a 2 min rest between sets + 30 min (15 walk + 15 at half the number of reps of low-intensity resistance training with free weights) + pacing for breathing			3
	CG: CON	Medical only.				
Fukuoka et al., 2016 [63]	IG: PR+ AMBMT	Exercise training, educational programs (5 education sessions), lung physiotherapy, and nutrition counseling + laughter yoga (deep breathing + hand clapping + laughter activities + cool down by deep breathing.	Exercise training + 10 min laughter yoga	Low to medium intensity	2	
	IG: PR	Exercise training, educational programs (5 education sessions), lung physiotherapy, and nutrition counseling.				
Hansen et al., 2019 [64]	IG: HMPR	Pulmonary telerehabilitation: supervised program (warm-up + END training + education session.	Weekly exercise volume 105 min (35 min exercise sessions + 5 min rest before beginning education session of 20 min (weekly education volume 60 min)		10	2
	IG: PR	Exercise training (warm-up + END + resistance training + cooldown) + education sessions.	Weekly exercise volume 120 min + Education sessions lasted 60 to 90 min		10. In one hospital (12)	2
Hagag et al., 2019 [65]	IG: PILATES	Pilates exercises: Warm-up (breathing, arm circles, hip rolls) + pilates phase (5 types of exercise) + cooldown (stretching)	7–10 min warm up + 40 min pilates (5 types of exercises, 7–8 min e/e) + 7–10 min cool-down	Warm-up: 9–11 Borg scale. Pilates phase: moderate intensity at 12–14 Borg scale + Cool down phase: 9–11 Borg scale (20)	12	3
	CG: CON	DB.	DB: 2 sets for 5 reps with rest intervals of 5–6 tidal breaths between exercises		12	3
Wen et al., 2020 [66]	IG: PR	END (CYC) + breathing (DB, PLB, chest breathing exercise, relaxation, cough training, and resistance breath training + walk + stair training + horizontal bar training.	CYC (medium speed, rest for 1 min after every 4 min of exercise, 15 min/day) + 15 min/day breathing exercises + 500 m walk (twice daily) + 10 min (twice daily) 5-step stair + 15 min (twice daily) horizontal bar training		4	twice daily
	CG: CON					
Kilic et al., 2021 [67]	IG: PR	Warm-up + ST upper and lower extremities + DB + stretches + cooldown + booklet and CD.	45 min exercise + 15 min of rest in each session. 10 min warm + 20 min ST exercises (10 times e/e) + 5 min DB (10 times e/e) + stretches (10 s e/e) + 10 min cooldown		12	3
	IG: HMPR	ST + respiratory exercises + CD and training booklet for learning reinforcement + checked by the researcher via phone.	45 min home exercises			3
Kantatong et al., 2019 [68]	IG: AMBMT	TCQ programme: 8 forms modified 3 times a week in the center-based programme led by a TCQ instructor + 2 days practise at home + poster to simplify self-practise at home.	Full breathing in supine or sitting position 4 times/day, 8–10 breaths at a time; increased the number of rounds up to 10 per day and performed it while sitting, standing, or walking		24 (12 at center + 12 at home)	3 at center + 2 at home
	CG: CON	Usual care without another intervention.				
Babkina et al., 2017 [69]	IG: AMBMT	Full yogic breathing intervention (deep, slow breathing: abdominal, thoracic, and clavicular) + diaries.				
	CG: CON	Usual care.				
Pradella et al., 2015 [70]	IG: HMPR	A week at the rehabilitation center learning the exercises and received printed material (educational booklet) with exercises to be performed (warm-up, aerobic activity, stretching, and relaxation) + received a log to record their activities + weekly telephone calls.	Warm-up included five 1 min exercises with a 1 min rest interval between exercises. Walk 40 min along a corridor or a street, climbing stairs 15 min, and exercising the arms with an oil can (1 kg) using diagonal movements for 15 min	Heart rate of 60–70% of MHR	8	3
	CG: CON	Weekly telephone calls.				
Kraemer et al., 2021 [71]	IG: AMBMT	Five core TCQ movements + four mind–body breathing techniques were integrated throughout the interventions + tai chi warm-up and cool-down exercises (1 h) + practice at home 3 times/week 30 min + 45 min DVD and audio file to encourage this home practice.	2 times/week 1 h + 3 times/week 30 min at home		12 + 12	2 + 1 s 12 weeks + 3 at home
	CG: CON	Four mind–body breathing techniques + mindful awareness of breath was emphasized in each of the techniques (1 h). Were also encouraged to practice at home three additional times per week for 30 min + 45-min DVD and audio files to facilitate home practice.	2 times/week 1 h + 3 times/week 30 min at home		12	2 + 3 at home
Barakat et al.,2008 [72]	IG: PR	PR: 30 min education and exercises (5 min warm-up + 10 min aerobic activity: diagonal arm raises, arm abd into elevation and reverse, arm abd, forward flexion, and reverse and straight leg rises) + 15 min cooldown) + 30 min cycling + dietary assessment and advice.	3 times/week 1 h	25 watts and increased the intensity by 5 watts each week until 40 watts. The aim was to reach the 80% of VO2 max of each patient	14	3
	CG: CON	Usual medical care.				

**Interventions and abbreviatures:** COMB: combined strength + endurance; ST: strength training; END: endurance training; CON: control; PR: pulmonary rehabilitation program; AC: arm crank ergometer; AWM: arm weight machine; LWM: leg weight machine; SC: stationary cycle; S: step-ups; Sq: wall squats; S/S: sit to stand exercises; UAE: upper limb unsupported arm exercises; Walk: walking; WLE: weight lifting exercise; CYC: cycling; TMW: treadmill walking; AMBMT: active mind–body movement therapies; TCQ: tai chi; PLB: pursed-lip breathing; DB: diaphragmatic breathing; IMT: inspiratory muscle training; QYJJ: qigong Yi Jinjing; LQG: Liuzijue qigong; HQG: health qigong; MHR: maximum heart rate; e/e: each exercise; max: maximal; RS: reinforcement sessions; min: minutes; rep: repetitions; IG: Intervention group; CG: control group.

**Table 3:** Pooled mean differences of physical activity on 6 MWT. Upper right triangle provides the pooled mean differences from pairwise comparisons (column intervention relative to row), lower left triangle pooled mean differences from the network meta-analysis (row intervention relative to column).

CONTROL	0.59 (0.31, 0.88)	0.54 (−0.13, 1.21)	0.86 (0.11, 1.61)	0.51 (0.19, 0.83)	1.97 (1.20, 2.75)	0.56 (0.24, 0.88)	NA	NA	1.10 (0.64, 1.56)
0.96 (0.61, 1.31)	AMBMT	−0.14 (−1.46,1.19)	NA	0.69 (0.12, 1.27)	NA	0.06 (−0.38, 0.50)	NA	NA	NA
0.90 (0.07, 1.74)	−0.06 (−0.94, 0.82)	COMB	0.07 (−0.65, 0.79)	NA	NA	0.22 (−0.44, 0.89)	NA	NA	NA
1.04 (−0.22, 2.29)	0.07 (−1.22, 1.37)	0.13 (−1.13, 1.40)	END	NA	NA	NA	NA	NA	NA
0.55 (0.17, 0.92)	−0.42 (−0.86, 0.02)	−0.36 (−1.26, 0.54)	−0.49 (−1.80, 0.81)	HM PR	NA	0.08 (−0.11, 0.26)	NA	0.02 (−0.54, 0.59)	NA
1.32 (0.18, 2.45)	0.35 (−0.84, 1.54)	0.41 (−1.00, 1.82)	0.28 (−1.42, 1.97)	0.77 (−0.43, 1.97)	PILATES	NA	NA	NA	NA
0.91 (0.52, 1.31)	−0.05 (−0.55, 0.45)	0.01 (−0.87, 0.89)	−0.12 (−1.43, 1.18)	0.37 (−0.11, 0.85)	−0.40 (−1.61, 0.80)	PR	0.06 (−0.22, 0.33)	NA	0.24 (−0.23, 0.70)
0.81 (−0.43, 2.06)	−0.15 (−1.43, 1.13)	−0.09 (−1.56, 1.38)	−0.22 (−1.98, 1.54)	0.27 (−1.01, 1.55)	−0.50 (−2.19, 1.19)	−0.10 (−1.28, 1.08)	PR + AMBMT	NA	NA
0.44 (−1.13, 2.01)	−0.52 (−2.11, 1.07)	−0.46 (−2.23, 1.31)	−0.59 (−2.60, 1.42)	−0.10 (−1.63, 1.42)	−0.87 (−2.81, 1.07)	−0.47 (−2.07, 1.13)	−0.37 (−2.36, 1.62)	PR + HM PR	NA
1.50 (0.46, 2.55)	0.54 (−0.55, 1.63)	0.60 (−0.72, 1.92)	0.47 (−1.16, 2.09)	0.96 (−0.13, 2.05)	0.19 (−1.36, 1.73)	0.59 (−0.46, 1.63)	0.69 (−0.89, 2.27)	1.06 (−0.82, 2.94)	PR + URBAN

CON: control; AMBMT: active mind–body movement therapies; COMB: combined; END: endurance; HMPR: home pulmonary rehabilitation program; PR: pulmonary rehabilitation program; PR + AMBMT: pulmonary rehabilitation program + active mind–body movement therapies; PR + HMPR: pulmonary rehabilitation program + home pulmonary rehabilitation program; PR + URBAN: pulmonary rehabilitation program + urban training.

## Data Availability

Data are available upon request.

## Supplementary Materials

The following supporting information can be downloaded at: https://www.mdpi.com/article/10.3390/ijerph192114539/s1, Figure S1: Risk of bias of studies included as assessed with the RoB2 tool; Figure S2: Risk of bias assessed with RoB2 for included studies; Figure S3: Network of available comparisons between supervised and no supervised pulmonary rehabilitation and home pulmonary program interventions on exercise capacity. Size of node is proportional to number of trial participants, and thickness of continuous line connecting nodes is proportional to number of participants randomised in trials directly comparing the two treatments. **HMPR:** Home pulmonary rehabilitation program; **PR:** Pulmonary rehabilitation program; **1:** Supervised; **2:** No supervised; Figure S4: Cumulative rankogram for each physical activity intervention. **CON:** control; **AMBMT:** Active mind-body movement therapies; **COMB:** combined; **END:** endurance; **HMPR:** Home pulmonary rehabilitation program; **PR:** Pulmonary rehabilitation program; **PR+AMBMT:** Pulmonary rehabilitation program+ Active mind-body movement therapies; **PR+HMPR:** Pulmonary rehabilitation program+ Home pulmonary rehabilitation program; **PR+URBAN:** Pulmonary rehabilitation program+ Urban training; Figure S5: Cumulative rankogram for each pulmonary rehabilitation program/ home pulmonary rehabilitation program supervised versus no supervised **HMPR:** Home pulmonary rehabilitation program; **PR:** Pulmonary rehabilitation program; **1:** Supervised; **2:** No supervised; Figure S6: Funnel plot for comparison-specific pooled mean differences. **CON:** control; **AMBMT:** Active mind-body movement therapies; **COMB:** combined; **END:** endurance; **HMPR:** Home pulmonary rehabilitation program; **PR:** Pulmonary rehabilitation program; **PR+AMBMT:** Pulmonary rehabilitation program+ Active mind-body movement therapies; **PR+HMPR:** Pulmonary rehabilitation program+ Home pulmonary rehabilitation program; **PR+URBAN:** Pulmonary rehabilitation program+ Urban training. Table S1: Reporting Elements for Systematic Reviews that incorporate Network Meta-Analysis; Table S2: Search strategy for Medline database; Table S3: Quality grading of evidence (GRADE); Table S4: Data for assessment of transitivity assumption baseline characteristics; Table S5: Pooled mean differences of supervised versus no supervised on 6MWT. Upper right triangle gives the pooled mean differences from pairwise comparisons (column intervention relative to row), lower left triangle pooled mean differences from the network meta-analysis (row intervention relative to column); Table S6: Effectiveness ranking of physical activity interventions; Table S7: Effectiveness ranking of supervised versus no supervised interventions; Table S8: Sensitivity analysis by comparation groups; Table S9: Heterogeneity statistics for each pairwise comparison; Table S10: Publication bias for the different comparation groups.
