# Supplementary material for: Efficacy of Different Types of Physical Activity Interventions on Exercise Capacity in Patients with Chronic Obstructive Pulmonary Disease (COPD): A Network Meta-Analysis
Source: Int J Environ Res Public Health. 2022 Nov 5;19(21):14539. doi: 10.3390/ijerph192114539 (PMC9656092; doi:10.3390/ijerph192114539)
Supplement: Supplementary file 1 [file ijerph-19-14539-s001.zip › ijerph-1984679-supplementary.pdf]

SUPPLEMENTARY MATERIAL

Table S1. Reporting Elements for Systematic Reviews that incorporate Network Meta-Analysis**.**

Section/Topic	Item #	Checklist Item	Reported on Page #
**TITLE**			
Title	1	Identify the report as a systematic review *incorporating a network meta-analysis (or related form of meta-analysis).*	***1***
**ABSTRACT**			
Structured summary	2	Provide a structured summary including, as applicable:<br>**Background:** main objectives<br>**Methods:** data sources; study eligibility criteria, participants, and interventions; study appraisal; and *synthesis methods, such as network meta-analysis.*<br>**Results:** number of studies and participants identified; summary estimates with corresponding confidence/credible intervals; *treatment rankings may also be discussed. Authors may choose to summarize pairwise comparisons against a chosen treatment included in their analyses for brevity.*<br>**Discussion/Conclusions:** limitations; conclusions and implications of findings.<br>**Other:** primary source of funding; systematic review registration number with registry name.	***1***
**INTRODUCTION**			
Rationale	3	Describe the rationale for the review in the context of what is already known*, including mention of why a network meta-analysis has been conducted.*	***1***
Objectives	4	Provide an explicit statement of questions being addressed, with reference to participants, interventions, comparisons, outcomes, and study design (PICOS).	***2***
**METHODS**			
Protocol and registration	5	Indicate whether a review protocol exists and if and where it can be accessed (e.g., Web address); and, if available, provide registration information, including registration number.	***2***
Eligibility criteria	6	Specify study characteristics (e.g., PICOS, length of follow-up) and report characteristics (e.g., years considered, language, publication status) used as criteria for eligibility, giving rationale. *Clearly describe eligible treatments included in the treatment network and note whether any have been clustered or merged into the same node (with justification).*	***2***
Information sources	7	Describe all information sources (e.g., databases with dates of coverage, contact with study authors to identify additional studies) in the search and date last searched.	***2***
Search	8	Present full electronic search strategy for at least one database, including any limits used, such that it could be repeated.	***2-3 and table S2***
Study selection	9	State the process for selecting studies (i.e., screening, eligibility, included in systematic review, and, if applicable, included in the meta-analysis).	***3***
Data collection process	10	Describe method of data extraction from reports (e.g., piloted forms, independently, in duplicate) and any processes for obtaining and confirming data from investigators.	***3***
Data items	11	List and define all variables for which data were sought (e.g., PICOS, funding sources) and any assumptions and simplifications made.	***3***

**Geometry of the network**	**S1**	Describe methods used to explore the geometry of the treatment network under study and potential biases related to it. This should include how the evidence base has been graphically summarized for presentation, and what characteristics were compiled and used to describe the evidence base to readers.	***5***
Risk of bias within individual studies	12	Describe methods used for assessing risk of bias of individual studies (including specification of whether this was done at the study or outcome level), and how this information is to be used in any data synthesis.	***4,6***
Summary measures	13	State the principal summary measures (e.g., risk ratio, difference in means). *Also describe the use of additional summary measures assessed, such as treatment rankings and surface under the cumulative ranking curve (SUCRA) values, as well as modified approaches used to present summary findings from meta-analyses.*	***7***
Planned methods of analysis	14	Describe the methods of handling data and combining results of studies for each network meta-analysis. This should include, but not be limited to:<br>• *Handling of multi-arm trials;*<br>• *Selection of variance structure;*<br>• *Selection of prior distributions in Bayesian analyses; and*<br>• *Assessment of model fit.*	***7***
**Assessment of Inconsistency**	**S2**	Describe the statistical methods used to evaluate the agreement of direct and indirect evidence in the treatment network(s) studied. Describe efforts taken to address its presence when found.	***5,6***
Risk of bias across studies	15	Specify any assessment of risk of bias that may affect the cumulative evidence (e.g., publication bias, selective reporting within studies).	***6***
Additional analyses	16	Describe methods of additional analyses if done, indicating which were pre-specified. This may include, but not be limited to, the following:<br>• Sensitivity or subgroup analyses;<br>• Meta-regression analyses;<br>• *Alternative formulations of the treatment network; and*<br>• *Use of alternative prior distributions for Bayesian analyses (if applicable).*	***8***
**RESULTS†**			
Study selection	17	Give numbers of studies screened, assessed for eligibility, and included in the review, with reasons for exclusions at each stage, ideally with a flow diagram.	***Suplem. Table 1***
**Presentation of network structure**	**S3**	Provide a network graph of the included studies to enable visualization of the geometry of the treatment network.	***Figure 1***
**Summary of network geometry**	**S4**	Provide a brief overview of characteristics of the treatment network. This may include commentary on the abundance of trials and randomized patients for the different interventions and pairwise comparisons in the network, gaps of evidence in the treatment network, and potential biases reflected by the network structure.	***4***
Study characteristics	18	For each study, present characteristics for which data were extracted (e.g., study size, PICOS, follow-up period) and provide the citations.	***Table 3 Supplementary material***
Risk of bias within studies	19	Present data on risk of bias of each study and, if available, any outcome level assessment.	***6,7***
Results of individual studies	20	For all outcomes considered (benefits or harms), present, for each study: 1) simple summary data for each intervention group, and 2) effect estimates and confidence intervals. *Modified approaches may be needed to deal with information from larger networks.*	***6,7 and figure 2 and 3 supplementary material***

Synthesis of results	21	Present results of each meta-analysis done, including confidence/credible intervals. *In larger networks, authors may focus on comparisons versus a particular comparator (e.g. placebo or standard care), with full findings presented in an appendix. League tables and forest plots may be considered to summarize pairwise comparisons.* If additional summary measures were explored (such as treatment rankings), these should also be presented.	***Table 2 and Supplementary table 6***
**Exploration for inconsistency**	**S5**	Describe results from investigations of inconsistency. This may include such information as measures of model fit to compare consistency and inconsistency models, *P* values from statistical tests, or summary of inconsistency estimates from different parts of the treatment network.	***8***
Risk of bias across studies	22	Present results of any assessment of risk of bias across studies for the evidence base being studied.	***6***
Results of additional analyses	23	Give results of additional analyses, if done (e.g., sensitivity or subgroup analyses, meta-regression analyses*, alternative network geometries studied, alternative choice of prior distributions for Bayesian analyses,* and so forth).	***8***
**DISCUSSION**			
Summary of evidence	24	Summarize the main findings, including the strength of evidence for each main outcome; consider their relevance to key groups (e.g., healthcare providers, users, and policy-makers).	***8***
Limitations	25	Discuss limitations at study and outcome level (e.g., risk of bias), and at review level (e.g., incomplete retrieval of identified research, reporting bias). *Comment on the validity of the assumptions, such as transitivity and consistency. Comment on any concerns regarding network geometry (e.g., avoidance of certain comparisons).*	***10***
Conclusions	26	Provide a general interpretation of the results in the context of other evidence, and implications for future research.	***11***
**FUNDING**			
Funding	27	Describe sources of funding for the systematic review and other support (e.g., supply of data); role of funders for the systematic review. This should also include information regarding whether funding has been received from manufacturers of treatments in the network and/or whether some of the authors are content experts with professional conflicts of interest that could affect use of treatments in the network.	***No funding***

PICOS = population, intervention, comparators, outcomes, study design.

* Text in italics indicateS wording specific to reporting of network meta-analyses that has been added to guidance from the PRISMA statement.

† Authors may wish to plan for use of appendices to present all relevant information in full detail for items in this section.

Table S2. Search strategy for Medline database.

"COPD"<br><br>OR<br><br>"Chronic obstructive Pulmonary disease”	**AND**	"Physical activity"<br>OR<br><br>“Pulmonary rehabilitation”<br>OR<br><br>Yoga<br>OR<br><br>“Active mind-body movements Therapies”<br>OR<br><br>“Aerobic exercise”<br>OR<br><br>“Exercise capacity”<br>OR<br><br>“6MWT”

Table S3. Quality grading of evidence (GRADE)

Certainty assessment						№ of patients		Effect	
**№ of studies**	**Study design**	**Risk of bias**	**Inconsistency**	**Indirectness**	**Imprecision**	**Intervention**	**Control**	**Relative (95% CI)**	**Importance**
**AMBMT versus COMB**									
1	randomised trials	serious	not serious	not serious	serious[a]	8/11 (72.7%)	3/11 (27.3%)	**-0.14** (-1.46 to 1.19)	NOT IMPORTANT
**AMBMT versus CON**									
14	randomised trials	not serious	not serious	not serious	not serious	540/1035 (52.2%)	495/1035 (47.8%)	**0.53** (0.39 to 0.67)	CRITICAL
**AMBMT vs HM PR**									
4	randomised trials	not serious	serious[b]	not serious	not serious	245/489 (50.1%)	244/489 (49.9%)	**0.55** (0.36 to 0.73)	IMPORTANT
**AMBMT vs PR**									
1	randomised trials	not serious	serious[b]	not serious	serious[a]	51/83 (61.4%)	32/83 (38.6%)	**0.06** (-0.38 to 0.50)	NOT IMPORTANT
**COMB vs CON**									
2	randomised trials	serious[c]	not serious	not serious	not serious	29/58 (50.0%)	29/58 (50.0%)	**0.54** (0.01 to 1.07)	IMPORTANT
**COMB vs END**									
1	randomised trials	serious[c]	not serious	not serious	serious[a]	15/30 (50.0%)	15/30 (50.0%)	**0.07** (-0.65 to 0.79)	NOT IMPORTANT
**COMB vs PR**									
1	randomised trials	serious	not serious	not serious	serious[a]	21/36 (58.3%)	15/36 (41.7%)	**0.22** (-0.44 to 0.89)	NOT IMPORTANT
**END vs CON**									
1	randomised trials	serious[c]	not serious	not serious	serious[a]	15/30 (50.0%)	15/30 (50.0%)	**0.86** (0.11 to 1.61)	IMPORTANT
**HM PR vs CON**									
10	randomised trials	serious[c]	not serious	not serious	not serious	384/714 (53.8%)	330/714 (46.2%)	**0.31** (0.16 to 0.47)	CRITICAL

Table S3. Quality grading of evidence (GRADE). (Continue)

Certainty assessment						№ of patients		Effect	Importance
**№ of studies**	**Study design**	**Risk of bias**	**Inconsistency**	**Indirectness**	**Imprecision**	**Intervention**	**Control**	**Relative (95% CI)**	
**Pilates vs CON**									
1	randomised trials	not serious	not serious	not serious	serious[a]	19/38 (50.0%)	19/38 (50.0%)	**1.97** (1.20 to 2.75)	IMPORTANT
**PR+AMBMT vs PR**									
2	randomised trials	serious[c]	serious[b]	not serious	not serious	99/200 (49.5%)	101/200 (50.5%)	**0.06** (-0.22 to 0.33)	NOT IMPORTANT
**PR+HM PR vs HM PR**									
1	randomised trials	serious[c]	serious[b]	not serious	serious[a]	24/48 (50.0%)	24/48 (50.0%)	**0.02** (-0.54 to 0.59)	NOT IMPORTANT
**PR+URBAN vs CON**									
1	randomised trials	serious[c]	not serious	not serious	serious[a]	34/88 (38.6%)	54/88 (61.4%)	**1.10** (0.64 to 1.56)	IMPORTANT
**PR+URBAN vs PR**									
1	randomised trials	serious[c]	not serious	not serious	serious[a]	34/71 (47.9%)	37/71 (52.1%)	**0.24** (-0.23 to 0.70)	IMPORTANT
**PR vs CON**									
11	randomised trials	serious[c]	not serious	not serious	not serious	287/584 (49.1%)	297/584 (50.9%)	**0.51** (0.33 to 0.69)	CRITICAL
**PR vs HM PR**									
3	randomised trials	not serious	not serious	not serious	not serious	220/444 (49.5%)	224/444 (50.5%)	**0.08** (-0.11 to 0.26)	IMPORTANT

AMBMT: Active mind-body movement therapies; **COMB:** Combined; **CON:** Control. **HMPR:** Home pulmonary rehabilitation program; **PR:** Pulmonary rehabilitation program; **END:** Endurance; **PR+AMBMT:** Pulmonary rehabilitation program+ Active mind-body movement therapies; **PR+HMPR:** Pulmonary rehabilitation program+ Home pulmonary rehabilitation program; **PR+URBAN:** Pulmonary rehabilitation program+ Urban training. **CI:** confidence interval

Explanations: a. We have only found one article comparing these two interventions. The sample is small. b. The results obtained in the meta-analysis and in the network do not go in the same direction. c. The proportion of information from studies with a high risk of bias is sufficient to affect the interpretation of the outcomes.

Table S4. Data for assessment of transitivity assumption baseline characteristics

Intervention		Age	Women	BMI	FEV1	MWT
AMBMT	N	16	5	9	14	16
	Mean	65,7881	11,9600	22,1044	55,1850	355,2613
	Minimum	52,50	1,00	18,89	37,13	219,10
	Maximum	72,20	21,60	25,50	74,43	593,50
COMB	N	4	0	3	4	4
	Mean	66,1750	-	27,0333	51,3000	379,8500
	Minimum	58,70	-	26,00	39,50	215,10
	Maximum	73,00	-	28,10	64,30	502,30
CON	N	32	10	20	29	32
	Mean	65,1703	17,8600	25,1320	48,2355	343,7884
	Minimum	42,09	5,60	19,97	23,21	83,79
	Maximum	73,60	41,00	31,00	75,31	470,00
END	N	1	0	1	1	1
	Mean	61,0000	-	24,0000	53,2000	224,1000
	Minimum	61,00	-	24,00	53,20	224,10
	Maximum	61,00	-	24,00	53,20	224,10
HMPR	N	15	10	11	15	15
	Mean	67,2313	28,1900	25,3364	47,4247	332,2820
	Minimum	56,96	4,30	19,90	29,44	88,79
	Maximum	73,60	54,00	28,40	62,40	485,10
PILATES	N	1	0	1	1	1
	Mean	43,0500	-	32,4000	62,7200	465,0000
	Minimum	43,05	-	32,40	62,72	465,00
	Maximum	43,05	-	32,40	62,72	465,00
PR	N	17	8	13	15	17
	Mean	67,1459	25,6375	25,2062	46,0627	361,9035
	Minimum	59,37	7,40	19,80	28,00	261,00
	Maximum	77,00	58,00	29,80	75,31	466,70
PR+ AMBMT	N	2	1	2	1	2
	Mean	74,3800	6,4000	22,0600	56,4000	324,2500
	Minimum	74,16	6,40	21,70	56,40	312,10
	Maximum	74,60	6,40	22,42	56,40	336,40
PR+ HMPR	N	1	0	1	1	1
	Mean	65,0000	-	25,0000	57,0000	463,0000
	Minimum	65,00	-	25,00	57,00	463,00
	Maximum	65,00	-	25,00	57,00	463,00
PR+ URBAN	N	1	0	1	1	1
	Mean	70,2000	-	27,2000	32,0000	358,8000
	Minimum	70,20	-	27,20	32,00	358,80
	Maximum	70,20	-	27,20	32,00	358,80

AMBMT: Active mind-body movement therapies; **COMB:** Combined; **CON:** Control. **HMPR:** Home pulmonary rehabilitation program; **PR:** Pulmonary rehabilitation program; **END:** Endurance; **PR+AMBMT:** Pulmonary rehabilitation program+ Active mind-body movement therapies; **PR+HMPR:** Pulmonary rehabilitation program+ Home pulmonary rehabilitation program; **PR+URBAN:** Pulmonary rehabilitation program+ Urban training.

Table S5. Pooled mean differences of supervised versus no supervised on 6MWT. Upper right triangle gives the pooled mean differences from pairwise comparisons (column intervention relative to row), lower left triangle pooled mean differences from the network meta-analysis (row intervention relative to column).

**CONTROL**						
0.84<br>(0.33, 1.36)	**HMPR1**					
0.58<br>(0.11, 1.05)	-0.26<br>(-0.87, 0.35)	**HMPR2**				
0.85<br>(0.46, 1.23)	0.00<br>(-0.55, 0.56)	0.27<br>(-0.30, 0.83)	**PR1**			
0.47<br>(-1.25, 2.20)	-0.37<br>(-2.13, 1.40)	-0.11<br>(-1.76, 1.55)	-0.37<br>(-2.12, 1.38)	**PR1+HMPR1**		
0.48<br>(-1.00, 1.95)	-0.36<br>(-1.89, 1.16)	-0.10<br>(-1.50, 1.30)	-0.37<br>(-1.87, 1.14)	0.01<br>(-0.88, 0.89)	**PR1+HMPR2**	
0.83<br>(-0.61, 2.27)	-0.01<br>(-1.51, 1.48)	0.25<br>(-1.25, 1.74)	-0.02<br>(-1.40, 1.37)	0.35<br>(-1.88, 2.59)	0.35<br>(-1.70, 2.40)	**PR2**

HMPR: Home pulmonary rehabilitation program; **PR:** Pulmonary rehabilitation program; **1:** Supervised; **2:** No supervised

Table S6. Effectiveness ranking of physical activity interventions.

**Intervention**	**Rank statistics**			**Probabilities**	
	**Mean**	**Median**	**95% CI**	**Best**	**SUCRA**
CON	7.60	9.50	(1, 10)	0.00	5.3
AMBMT	5.30	6.00	(1, 10)	1.1	59.3
COMB	3.90	3.50	(2, 6)	3.7	53.7
END	4.30	4.00	(3, 6)	14.1	59.5
HMPR	6.00	8.00	(1, 9)	0.0	28.8
Pilates	5.50	6.00	(2, 9)	27.2	73.6
PR	5.30	6.50	(1, 9)	0.3	55.4
PR + AMBMT	4.40	4.00	(2, 7)	8.2	48.7
PR+ HMPR	5.70	6.50	(2, 8)	5.6	33.3
PR + URBAN	6.40	7.50	(1, 10)	39.8	82.5

CON: Control; **AMBMT:** Active mind-body movement therapies; **COMB:** Combined; **END:** Endurance; **HMPR:** Home Pulmonary Rehabilitation; **PR:** Pulmonary Rehabilitation; **HMPR:** Home Pulmonary Rehabilitation; **URBAN:** Urban training

Table S7. Effectiveness ranking of supervised versus no supervised interventions.

**Intervention**	**Rank statistics**			**Probabilities**	
	**Mean**	**Median**	**95% CI**	**Best**	**SUCRA**
CON	4.71	7.0	(1, 7)	0.00	11.7
HMPR1	3.57	2.0	(2, 6)	19.8	69.0
HMPR2	3.57	3.0	(1, 6)	2.4	47.4
PR1	3.86	4.0	(1, 7)	15.7	69.7
PR1+HMPR1	4.14	4.0	(2, 6)	17.5	45.2
PR1+HMPR2	3.43	3.0	(1, 5)	9.8	44.8
PR2	4.43	5.0	(1, 7)	34.8	62.0

CON: Control; **HMPR1:** Supervised Home pulmonary rehabilitation; **HMPR2:** No Supervised Home pulmonary rehabilitation program; **PR1:** Supervised Pulmonary rehabilitation program; **PR2:** No Supervised Pulmonary rehabilitation program

Table S8. Sensitivity analysis by comparation groups

Active mind-body movement therapies (AMBMT) vs Control group

Reference	ES	LL	UL
Babkina et al., 2017	0.572	0.264	0.880
Chan et al., 2011	0.623	0.310	0.935
Chan et al., 2013	0.623	0.319	0.935
Donesky-Cuenco et al., 2009	0.618	0.319	0.917
Gu et al., 2012	0.599	0.291	0.907
Gupta et al., 2014	0.625	0.323	0.927
Kantatong et al., 2019	0.567	0.267	0.867
Kraemer et al., 2021	0.630	0.326	0.934
Liu et al., 2012	0.572	0.266	0.878
Ng et al., 2011	0.612	0.303	0.922
Niu et al., 2014	0.607	0.304	0.911
Ranjita et al., 2016	0.608	0.299	0.917
Yeh et al., 2010	0.607	0.313	0.901
Zhang et al., 2016	0.453	0.304	0.602

AMBMT vs Home Pulmonary Rehabilitation (HM PR)

Reference	ES	LL	UL
Chan et al., 2011	0.848	0.525	1.644
Chan et al., 2013	0.848	0.525	1.644
Xiao et al., 2015	0.753	-0.082	1.588
Zhang et al., 2016	0.355	0.158	0.552

Combined vs Control groups

Reference	ES	LL	UL
Daabis et al., 2016	0.132	-0.384	0.647
Simpson et aL.,1992	0.469	-0.328	1.266
Daabis et al., 2016	0.539	-0.133	1.211

HM PR vs Control groups

Reference	ES	LL	UL
Chan et al., 2011	0.586	0.227	0.944
Chan et al., 2013	0.586	0.277	0.944
De Sousa Pinto et al., 2014	0.456	0.126	0.786
Ghanem et al., 2010	0.330	0.101	0.559
Muñoz Fernández et al., 2009	0.505	0.157	0.853
Nguyen et al., 2013	0.566	0.202	0.930
Nguyen et al., 2013	0.567	0.205	0.930
Oh., 2003	0.489	0.151	0.827
Pradella et al., 2016	0.518	0.166	0.869
Zhang et al., 2016	0.500	0.144	0.857

Pulmonary Rehabilitation (PR) vs Control groups

Reference	ES	LL	UL
Barakat et al., 2008	0.475	0.178	0.772
Borghi-Silva et al., 2009	0.546	0.200	0.892
Engstrom et al., 1999	0.585	0.237	0.933
Gottlieb et al., 2011	0.578	0.230	0.926
Liu et al., 2012	0.530	0.177	0.884
Pleguezuelos et al., 2013	0.525	0.170	0.880
Román et al., 2013	0.630	0.310	0.950
Román et al., 2013	0.651	0.351	0.951
Singh et al., 2003	0.592	0.249	0.935
Theander et al., 2009	0.585	0.245	0.925
Wen et al., 2020	0.496	0.169	0.823

ES: Effect size; **LL:** Low limit; **UL:** Upper limit

Table S9. Heterogeneity statistics for each pairwise comparison.

Intervention	Q (df)	p	I^2	τ^2
AMBMT vs COMB	0.00	-	-	0.0000
AMBMT vs CON	61.28	0.000	78.8	0.2223
AMBMT vs HM PR	28.75	0.000	89.6	0.3080
AMBMT vs PR	0.00	-	-	0.0000
COMB vs CON	1.62	0.203	38.4	0.0902
COMB vs END	0.00	-	-	0.0000
COMB vs PR	0.00	-	-	0.0000
END vs CON	0.00	-	-	0.0000
HM PR vs CON	36.64	0.000	75.4	0.1903
Pilates vs CON	0.00	-	-	0.0000
PR + AMBMT vs PR	0.14	0.704	0.0	0.0000
PR + HM PR vs HM PR	0.00	-	-	0.0000
PR + URBAN vs CON	0.00	-	-	0.0000
PR + URBAN vs PR	0.00	-	-	0.0000
PR vs CON	34.70	0.000	71.2	0.2044
PR vs HM PR	1.15	0.564	0.0	0.0000

AMBMT: Active mind-body movement therapies; **COMB:** Combined; **CON:** Control. **HMPR:** Home pulmonary rehabilitation program; **PR:** Pulmonary rehabilitation program; **END:** Endurance; **PR+AMBMT:** Pulmonary rehabilitation program+ Active mind-body movement therapies; **PR+HMPR:** Pulmonary rehabilitation program+ Home pulmonary rehabilitation program; **PR+URBAN:** Pulmonary rehabilitation program+ Urban training.

Table S10. Publication bias for the different comparation groups

Comparation group	β	p
AMBMT vs CON	1.444	0.526
AMBMT vs HMPR	17.524	0.003
COMB vs CON	36.338	0.441
HMPR vs CON	5.034	0.004
PR vs CON	-5.584	0.139

AMBMT: Active mind-body movement therapies; **CON:** Control; **HMPR:** Home pulmonary rehabilitation program; **COMB:** Combined; **PR:** Pulmonary rehabilitation program

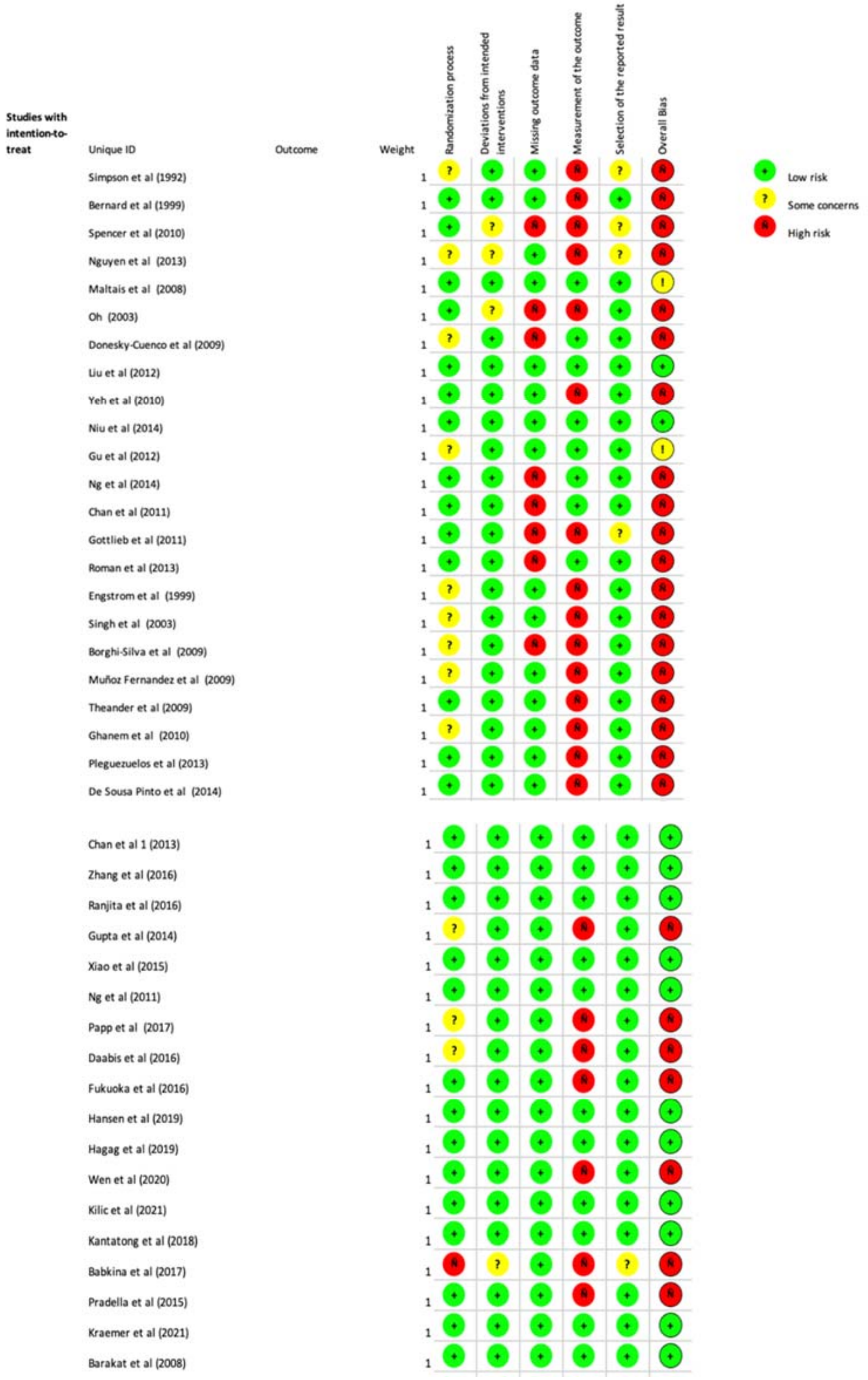

Figure S1. Risk of bias of studies included as assessed with the RoB2 tool.

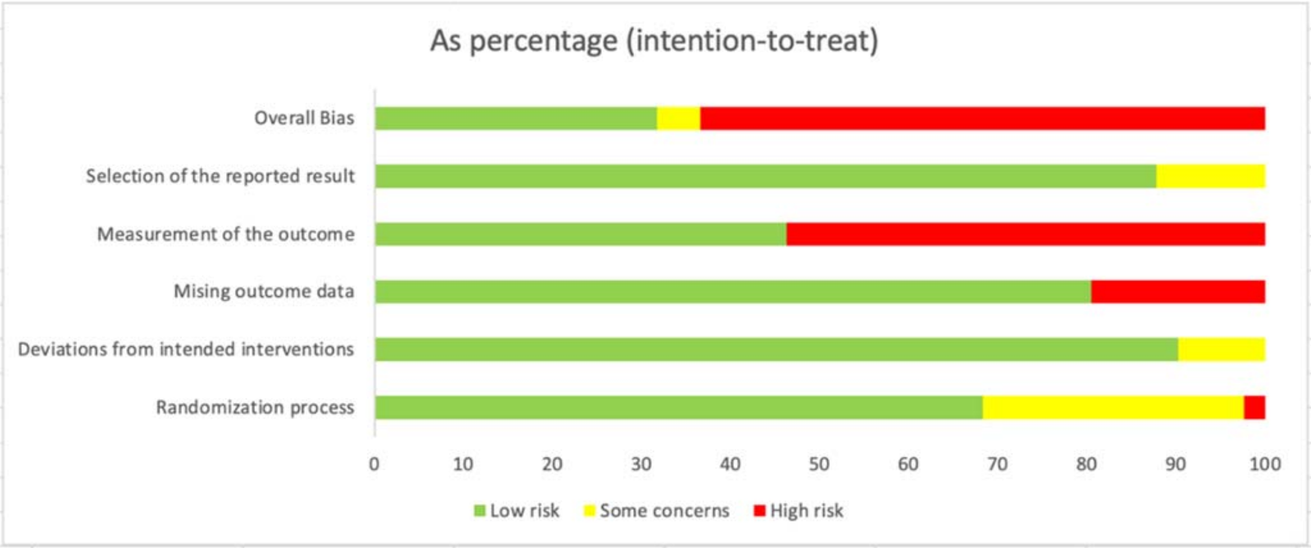

Figure S2. Risk of bias assessed with RoB2 for included studies.

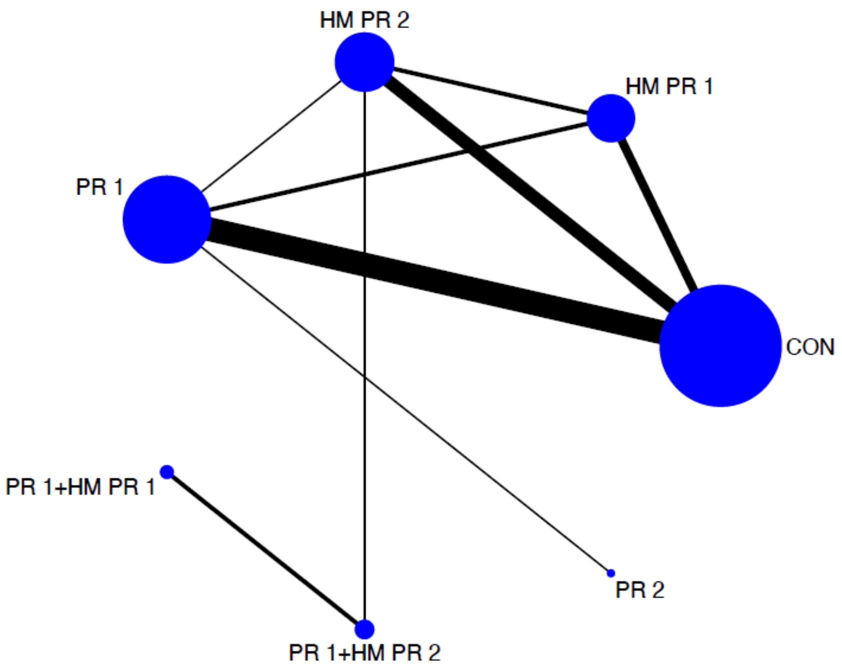

Figure S3. Network of available comparisons between supervised and no supervised pulmonary rehabilitation and home pulmonary program interventions on exercise capacity. Size of node is proportional to number of trial participants, and thickness of continuous line connecting nodes is proportional to number of participants randomised in trials directly comparing the two treatments. **HMPR:** Home pulmonary rehabilitation program; **PR:** Pulmonary rehabilitation program; **1:** Supervised; **2:** No supervised.

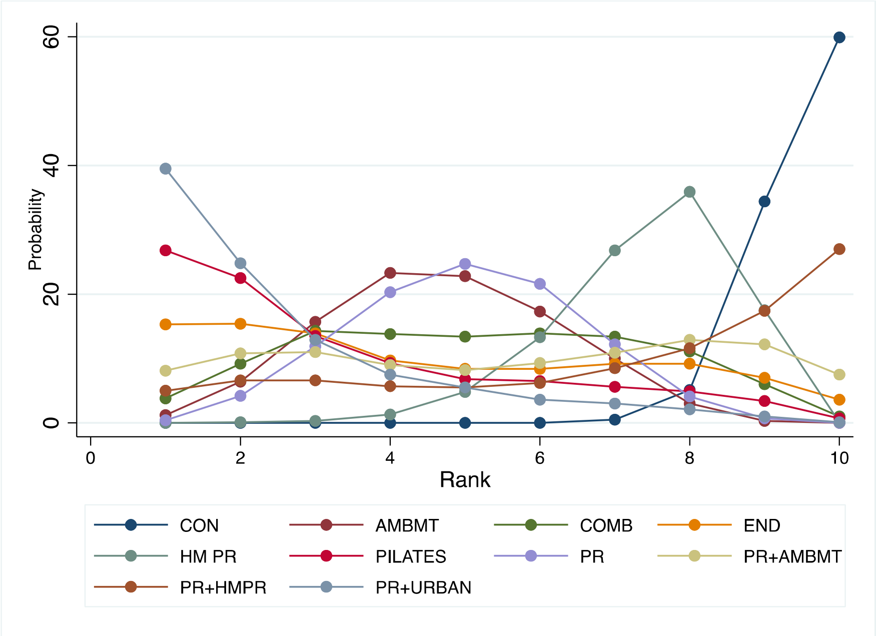

Figure S4. Cumulative rankogram for each physical activity intervention. **CON:** control; **AMBMT:** Active mind-body movement therapies; **COMB:** combined; **END:** endurance; **HMPR:** Home pulmonary rehabilitation program; **PR:** Pulmonary rehabilitation program; **PR+AMBMT:** Pulmonary rehabilitation program+ Active mind-body movement therapies; **PR+HMPR:** Pulmonary rehabilitation program+ Home pulmonary rehabilitation program; **PR+URBAN:** Pulmonary rehabilitation program+ Urban training.

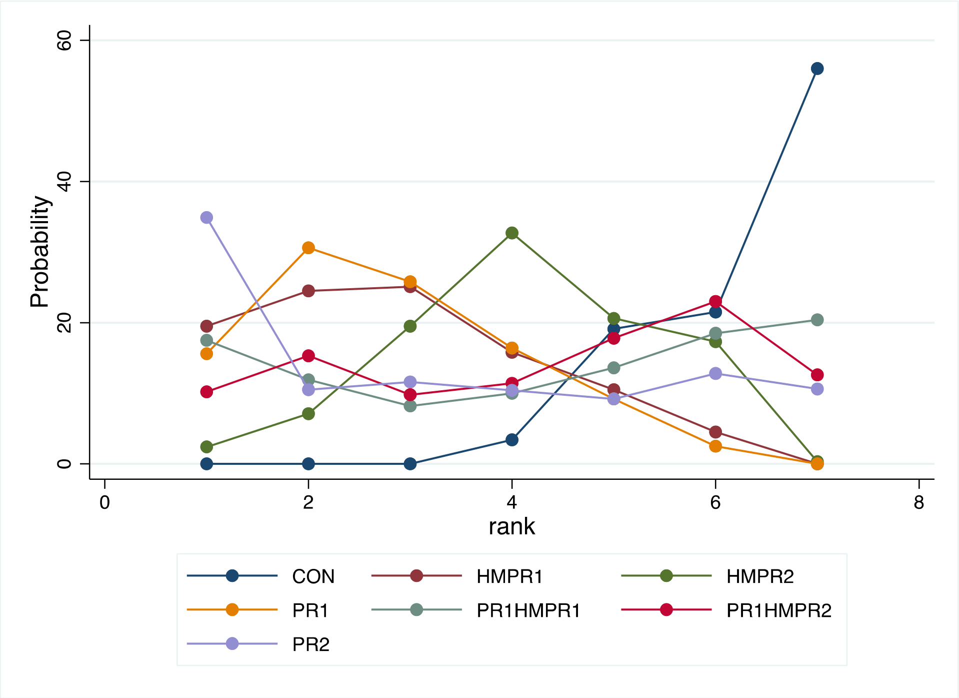

Figure S5. Cumulative rankogram for each pulmonary rehabilitation program/ home pulmonary rehabilitation program supervised versus no supervised **HMPR:** Home pulmonary rehabilitation program; **PR:** Pulmonary rehabilitation program; **1:** Supervised; **2:** No supervised.

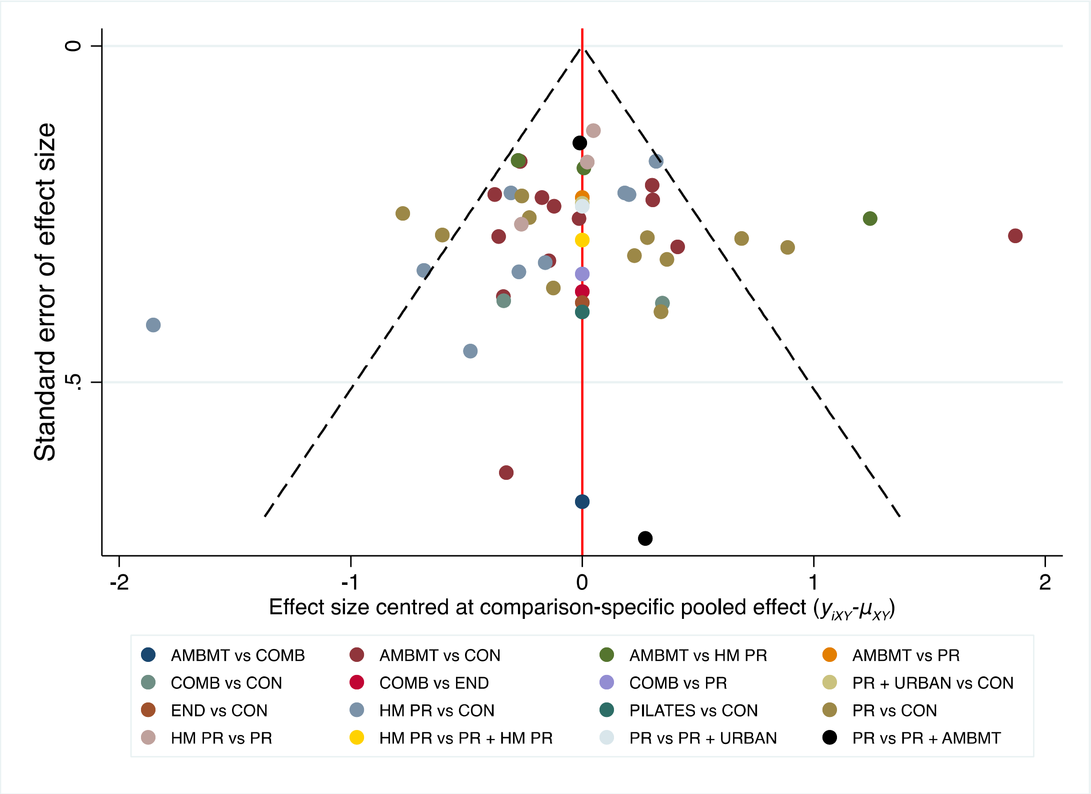

Figure S6. Funnel plot for comparison-specific pooled mean differences

CON: control; **AMBMT:** Active mind-body movement therapies; **COMB:** combined; **END:** endurance; **HMPR:** Home pulmonary rehabilitation program; **PR:** Pulmonary rehabilitation program; **PR+AMBMT:** Pulmonary rehabilitation program+ Active mind-body movement therapies; **PR+HMPR:** Pulmonary rehabilitation program+ Home pulmonary rehabilitation program; **PR+URBAN:** Pulmonary rehabilitation program+ Urban training.
